# Bioinspired Reversible Adhesive with High Strength for Wearable Electronics under Diverse Environments

**DOI:** 10.34133/research.1309

**Published:** 2026-06-04

**Authors:** Yihang Wu, Huiming Liu, Hongmiao Tian, Xiangming Li, Duorui Wang, Bo Sun, Quanyi Zhao, Jinyu Zhang, Tianxiang Lan, Guifang Liu, Xiaoliang Chen, Chunhui Wang, Jinyou Shao

**Affiliations:** ^1^ Micro-and Nano-technology Research Center, State Key Laboratory for Manufacturing Systems Engineering, Xi’an Jiaotong University, Xi’an, Shaanxi 710049, China.; ^2^ Frontier Institute of Science and Technology (FIST), Xi’an Jiaotong University, Xi’an, Shaanxi 710049, China.

## Abstract

Wearable electronics serve as critical tools for human health monitoring and equipment operation assessment. A fundamental prerequisite for their stable performance is the ability to attach flexibly and reliably to the surfaces of human bodies or equipment. However, existing adhesion methods for wearable devices face substantial challenges in simultaneously achieving high-strength reversible bonding and environmental adaptability across diverse conditions, hindering the practical applications in complex and variable environments. Here, inspired by the excellent adhesion behaviors of geckos and octopuses, we designed a dual bioinspired adhesive microstructure consisting of an annular stalk, a microdome, and an annular tip. The large-area precise formation of this complex 3-dimensional microstructure is achieved via simple imprinting and photolithography. The fabricated adhesive demonstrated reversible and superior normal adhesion in complex environments including vacuum (120 kPa), dry (201 kPa), moist (173 kPa), and underwater (165 kPa). In situ observations of the contact splitting reveal that its extraordinary performance derives from the unique crack reentry phenomenon across various environments, which effectively inhibits crack propagation and improves the adhesion forces. Simulations further clarify the causes of crack nucleation and the underlying adhesion mechanisms. Demonstrations of the adhesive in skin-attachable electronics across multiple environments highlight its potential for applications in wearable electronics operating under diverse and complex conditions, promoting rapid development of wearable flexible electronics.

## Introduction

Wearable electronics have been extensively utilized in the monitoring of human body conditions [1–5] and the motion capture of devices, including dexterous hands [6–8] or human-like robots [9–11]. They serve as a crucial approach for lightweight real-time human body monitoring and guaranteeing the operation of equipment. Among them, flexible skin-contact wearable technologies have attracted widespread attention because they can better conform to the morphology of human skin or equipment surfaces, thereby achieving faster responses and high-fidelity signals [12–14]. In addition to electrical characteristics, a critical prerequisite for the stable operation of such wearable electronics is their ability to adhere reliably and flexibly to target surfaces. More specifically, these devices must meet complex adhesion requirements: (a) high-strength and reversible adhesion, which enables them to attach to and detach from human skin or equipment surfaces on demand; and (b) effective adhesion performance across diverse environmental conditions, including dry, moist, underwater, and vacuum environments. Practical application interfaces, particularly those involving skin contact, are inherently complex because of the coupling of diverse environments. For example, to accommodate both dry and sweaty skin, wearable electronics must exhibit robust adhesion under both dry and wet conditions. Furthermore, for flexible skin-attached wearable electronics used in high-altitude environments or by astronauts, low-pressure adhesion capability must be considered. This is because ambient pressure decreases to approximately 60 kPa at an altitude of 4000 m, while the pressure within the space suit usually remains around 30 kPa [15]. Collectively, these multifaceted adhesion requirements impose stringent constraints and present significant challenges for the advancement of wearable electronic technologies.

Considerable research has been devoted to developing efficient and reliable attachment methods for wearable electronics, significantly advancing the development of this field. However, existing approaches still face challenges in meeting the practical demands of reversibility and adaptability to diverse operational environments. The attachment strategy based on chemical bonding, such as mussel-inspired chemically cross-linked adhesives, can offer moderate resistance to environmental disturbances [16–18]. However, such permanent bonding is inherently irreversible, conflicting with the need for flexible operation in wearable devices. In addition, it may cause damage not only to the device itself but also to the adhered surface when peeling. Alternative approaches using hydrogel-based adhesive layers enable partial reversibility [19,20]; however, these systems have the common problem that they are prone to losing stickiness due to water loss over time. Furthermore, under vacuum or low-pressure conditions, rapid solvent evaporation exacerbates instability, limiting their capacity for sustained functionality in complex and dynamic environments.

In recent years, attachment using bioinspired microstructured adhesives has emerged as a key trend in wearable electronics. This approach achieves interfacial adhesion purely through structural design, without relying on chemical cross-linking. It maximizes contact area and enables reversible, conformal adhesion to irregular surfaces such as skin or devices, thus ensuring reliable performance of flexible wearable systems [21]. For instance, wearable dry electrodes incorporating tree-frog-inspired hexagonal microstructures demonstrate robust skin adhesion, facilitating stable and long-term monitoring of various physiological electrical signals [22]. Similarly, wearable devices based on beetle-mimetic adhesion structures achieve adaptive attachment to the skin, enabling rapid, on-site collection of bodily fluid information [23]. Furthermore, multiplexed wearable systems utilizing beetle-inspired adhesion strategies have successfully realized simultaneous acquisition of multiple human physiological signals [24]. However, these studies are limited to simple dry/wet conditions, and it remains challenging to ensure effective adhesion in complex and diverse environments, such as those coupling low-pressure and moist conditions. Current attachment methods for wearable electronics have significantly hindered the advancement of the wearable electronics field.

**Fig. 1.**
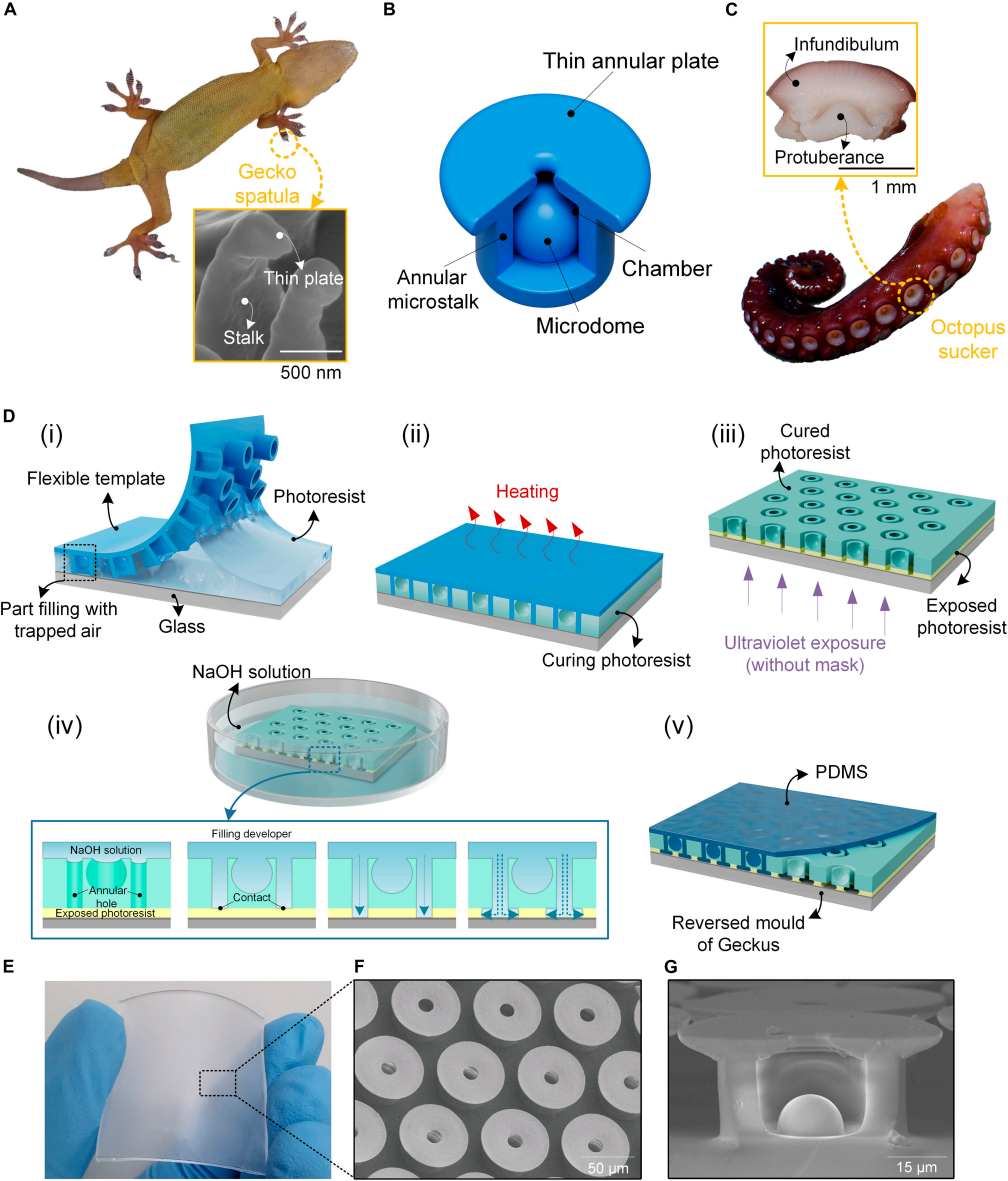
Design and fabrication of Geckus structure. (A) Image of a gecko. The inset is the scanning electron microscopy (SEM) image of gecko setae. (B) Illustration of a Geckus structure. (C) Image of an octopus tentacle. The inset is an image of the octopus sucker. (D) Fabrication process: (i) imprinting of the photoresist using a rolling press with a flexible template; (ii) curing of the patterned photoresist; (iii) back exposure of the cured photoresist; (iv) development. The inset shows the specific process of development. (v) Molding with polydimethylsiloxane (PDMS). (E) Image of a large-area Geckus patch. (F and G) SEM images of a Geckus array and of the cross-section of a single Geckus structure, respectively.

We believe that this limitation in application environments is primarily attributed to the singularity of the bionic model. Specifically, the attachment mechanism of a single biological organism is typically adapted to only one specific condition or basic dry and wet environments. Therefore, merely imitating a single biological attachment device is insufficient to fully meet practical demands, while coupling bionics may offer a viable strategy for overcoming these constraints. Fortunately, we have drawn important inspiration from the behaviors of 2 natural creatures. Geckos exhibit rapid climbing capabilities on vertical and inverted surfaces, demonstrating robust and reversible adhesion in dry environments [25]. This performance originates from arrays of spatula contact units on their foot pads [26], which consist of stalks and thin plate contact terminals (Fig. 1A). These spatulae can generate intermolecular interactions with foreign surfaces under intimate contact, generating substantial van der Waals forces. Notably, this adhesive mechanism retains its effectiveness under low-pressure conditions and even in vacuum environments. In contrast, octopuses achieve stable locomotion and precise object manipulation in aquatic settings through suction forces generated by the suckers on their arms [27], demonstrating robust and reversible adhesion under wet conditions. Furthermore, studies have identified that the sealing-maintained infundibulum and suction-assisted protuberance of the octopus suckers (Fig. 1C) are the key elements in the adsorption process [28,29]. Nevertheless, both biological systems present inherent limitations. The gecko’s van der Waals adhesion method is compromised in aqueous environments [30], while the suction effect used by octopus suckers fails or diminishes significantly under vacuum and low-pressure conditions. Therefore, integrating the advantages of both attachment systems while circumventing their respective limitations could offer a novel approach to addressing these issues. These issues also pose considerable challenges for the design and manufacture of such adhesive structures.

Herein, we present an incorporated 3-dimensional (3D) bionic adhesive architecture—the gecko- and octopus-inspired structure (Geckus; Fig. 1B). This structure consists of a cavity encircled by the annular microstalk, an embedded microdome in the chamber, and a thin annular plate equipped at the tip of the annular microstalk. This design realizes the integration of 2 functional architectures from gecko setae and octopus sucker at the micrometer scale. Among these features, the T-shaped architecture formed by the annular microstalk and the thin annular plate enables efficient van der Waals forces. The microdome in the chamber can facilitate capillary-assisted suction forces. In addition, the thin annular plate effectively seals the chamber to maintain the negative pressure inside. Collectively, these features endow it with strong and undiminished adhesion under vacuum, dry, moist, and underwater conditions, thus fulfilling the requirements for reversible operations in diverse environments. Notably, this structure is not merely a combination of 2 biomimetic elements but achieves a synergistic effect where the whole exceeds the sum of its parts, with its adhesion performance far surpassing that of the biological organisms.

The Geckus exhibits a complex 3D configuration, and achieving large-area, controllable array fabrication of such microscale structures remains a substantial technical challenge. Conventional manufacturing processes are insufficient to meet the processing demands of these intricate geometries, primarily due to the difficulty in controllably fabricating noncoplanar 3D features, including embedded spherical microdomes and flat annular plate tips. This paper presents a novel and ingenious fabrication strategy based on double patterning of photoresist, as illustrated in Fig. 1D. We creatively used a flexible template to imprint the flowing liquid photoresist, controlling incomplete filling to trap air and form spherical microdome features. Subsequently, the patterned and solidified photoresist was subjected to backside exposure to construct the latent image of the plate feature. This manufacturing strategy enables rapid and precise integrated molding of large-area Geckus arrays. It avoids the issues of excessive processing time and morphological distortion caused by material overcuring during point-by-point or layer-by-layer curing, which are common in conventional methods such as micro–nano 3D printing.

The experimental results show that the fabricated Geckus patch exhibits outstanding normal adhesion in vacuum (120 kPa), dry (201 kPa), moist (173 kPa), and underwater (165 kPa) environments. It also demonstrates considerable shear adhesion and peeling forces under these conditions. Observations of the contact splitting behaviors of Geckus under both dry and underwater conditions reveal that the nucleation modes and propagation directions of cracks serve as key contributors to the high adhesion strength across multiple environments. Finite element analysis elucidates the interfacial mechanical behavior, uncovering the underlying mechanisms of adhesion and crack nucleation. Finally, we used the Geckus patch to develop a sense–adhesion-integrated wearable electronic device, which can realize high-fit gesture sensing on moist skin under both atmospheric and low pressure (30 kPa). To the best of our knowledge, the research presented in this paper regarding the design, controllable fabrication, performance, and underlying mechanisms of adhesive structures stands at the international forefront. It presents methodologies for the reliable attachment of wearable electronics in complex and diverse environments, thereby providing a fundamental driving force for the advancement of this field.

## Results

### Fabrication method of the Geckus

The Geckus is a complex microscale 3D structure consisting of a thin-plate tip, a toroidal stalk, and a dome-like structure in a hollow chamber. Therefore, it is difficult for conventional micromanufacturing technologies to rapidly fabricate large-area array patches.

To achieve a fast, controllable array fabrication of Geckus patches, we adopted an ingenious method, which involves the imprinting and photoetching of photoresist (AZ P4620). Specifically, the manufacturing process consisted of 5 steps:

1. Photoresist imprinting. The photoresist was uniformly spin coated (500 rpm for 9 s and 700 rpm for 40 s) on a Cr-plated glass substrate with a 10-nm-thick Cr layer. The uncured photoresist was imprinted using a uniform rolling press with a flexible template, i.e., the hexagonal arrangement annular-hole patch based on polydimethylsiloxane (PDMS) with a height of 30 μm and inner and outer diameters of 30 and 50 μm, respectively [Fig. 1D(i)]. Here, PDMS was selected as the template material due to its flexibility, chemical stability, low surface energy, and good air permeability. These properties enable high-fidelity replication of microstructures without reacting with the photoresist, facilitate easy demolding, and allow solvent evaporation during thermal curing without swelling. The photoresist quickly fills the interconnected channels between the microholes when pressing down the template, while, at the same time, the photoresist is trapped and partly fills the blind microholes, forming dome-like shapes by wrapping the air remaining in the holes (Fig. S6). Since the initial photoresist layer obtained via spin coating has a uniform thickness and the mechanical pressure applied during imprinting is both uniform and constant, the amount of photoresist trapped in the blind microholes is highly reproducible. Consequently, the volume of trapped air remains consistent, ensuring uniform dome morphology. Throughout the entire imprinting process, a critical aspect is ensuring that the substrate surface is smooth and that the process is free from dust contamination. A rough substrate directly leads to nonuniform imprinted structures, while dust contamination may cause local structural defects. Both factors affect the microdome morphology. To evaluate the morphological uniformity of the microdomes fabricated by our technique, we prepared 3 batches of samples and randomly selected microdomes to measure their characteristic dimensions. The results showed that the average radius was 8.99 μm (standard deviation, 0.13 μm), and the average height was 11.73 μm (standard deviation, 0.19 μm). Figure S8 presents cross-sectional scanning electron microscopy (SEM) images of the 3 batches, revealing uniform microdome dimensions across all batches. These results demonstrate that the microdomes fabricated by our process exhibit excellent morphological uniformity and good batch-to-batch consistency, providing a structural foundation for stable adhesion performance.
2. Fixation of the imprinted geometric morphology. The sample was placed in an oven at 95 °C for 100 min [Fig. 1D(ii)]; the photoresist was solidified and tightly bonded to the glass substrate as a consequence of the volatilization of the organic solvent under heating.
3. Lithography to generate the latent image of the thin-plate tip [Fig. 1D(iii)]. After removing the flexible template, a structured photoresist pattern was obtained. On this basis, we performed a full-area maskless exposure from the backside of the sample to expose the bottom layer of the photoresist, with the thickness of the exposed layer determined by the exposure time.
4. Development of the latent image of the thin-plate tip [Fig. 1D(iv)]. The sample was fully immersed in the developer and subjected to vacuum treatment to fill the annular holes with the developer, allowing the developer to reach the exposed bottom photoresist layer through the annular holes. The developer gradually dissolved the exposed photoresist from both sides of the circular openings. Due to the isotropic nature of the photoresist development process, an inner and outer annular brim-like pattern was eventually formed, with the size of the brims controllable by the development time.
5. Molding [Fig. 1D(v)]. The PDMS base and curing agent were mixed at a 10:1 weight ratio and homogenized for 5 min under stirring to ensure uniform dispersion. The PDMS prepolymer was subsequently poured into the photoresist reverse mold of the Geckus structure and cured at 100 °C for 12 h to achieve full cross-linking. Since the photoresist is soluble in acetone, the reverse photoresist mold with the cured Geckus patch was immersed in acetone under ultrasonic agitation for 20 min. After the photoresist template was totally soluble and removed, the Geckus adhesive was completely demolded without constraints of the template. This method can ensure the damage-free demolding of this complex 3D structure.

The proposed fabrication approach can not only be used to prepare large-area (3.1 cm × 3.1 cm) Geckus arrays (Fig. 1E; with a thin-plate tip center-to-center distance of 80 μm) but also precisely control the morphology of the Geckus structure. Figure 1F shows the Geckus sample with a thin-plate tip inner diameter of 15 μm and a thin-plate tip outer diameter of 65 μm, and its cross-section is presented in Fig. 1G. The ability of this technology to precisely control the shape of the obtained Geckus structures is demonstrated by the preparation of Geckus samples with various tip sizes shown in Fig. S9.

### Adhesion performance of the Geckus in different environments

**Fig. 2.**
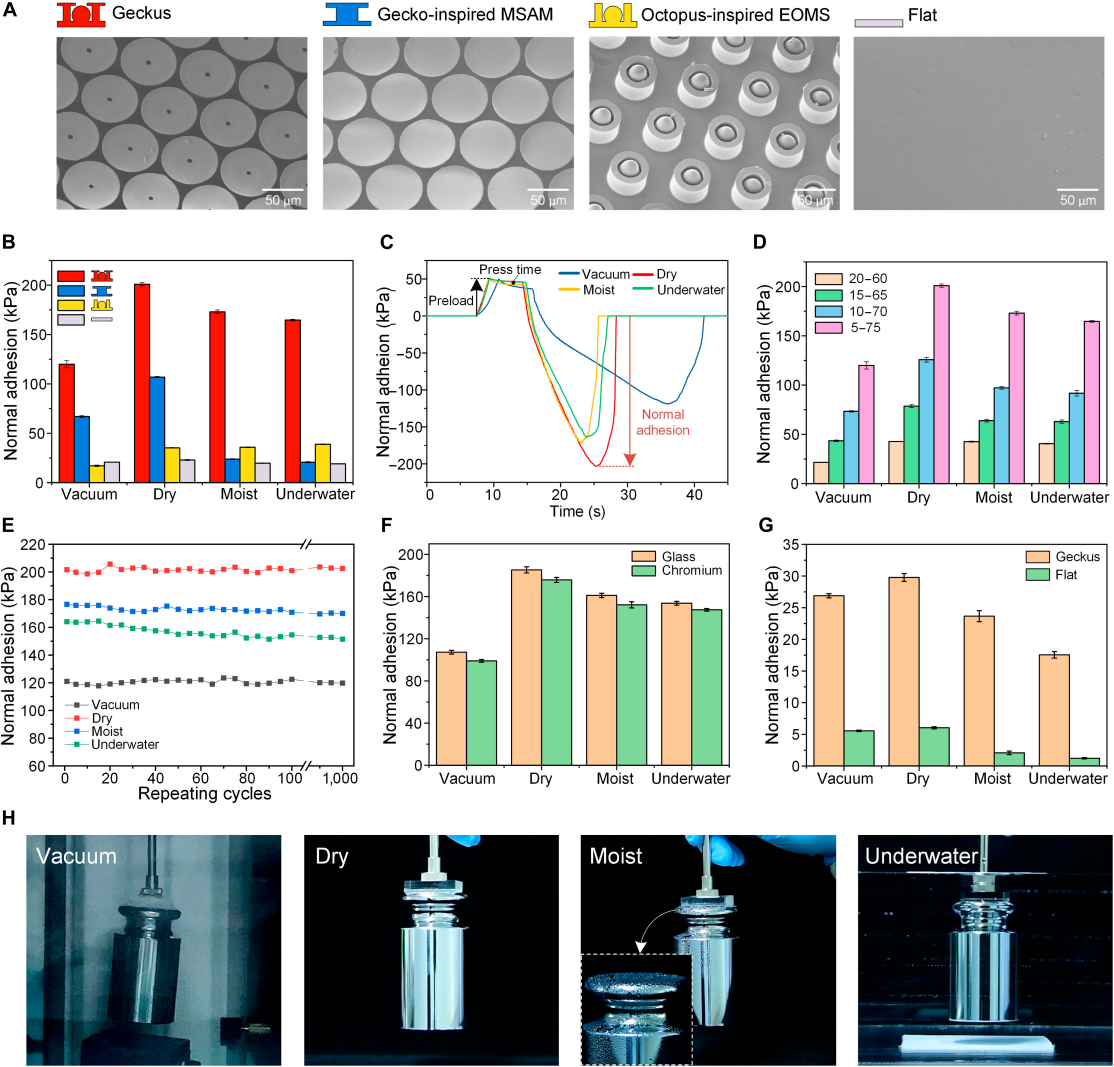
Characterizations of the adhesion forces under various environments and demonstrations of the grasping capacity of a Geckus patch under various environments. (A) Scanning electron microscopy (SEM) images of the Geckus, gecko-inspired mushroom-shaped adhesion microstructure (MSAM) structure, extruded-octopus-inspired microsucker (EOMS) structure, and flat patch. (B) Comparison of the normal adhesion strengths of the 4 different patches under various environments on a silicon surface (with a preload of 50 kPa). (C) Typical time-dependent adhesion profiles for the normal adhesion of Geckus in the measurements, with a preload of 50 kPa on a silicon surface under vacuum, dry, moist, and underwater conditions. (D) Comparison of the normal adhesion strengths of Geckus patches with different tip sizes under various environments on a silicon surface (with a preload of 50 kPa). (E) Normal adhesion data of the optimized Geckus under 1,000 repeat adhesion tests on a silicon surface under vacuum, dry, moist, and underwater conditions. (F) Normal adhesion forces of the optimized Geckus on different substrates (glass and chromium metal surfaces) under vacuum, dry, moist, and underwater conditions. (G) Comparisons of the normal adhesion on the rough goat skin between the optimized Geckus (tip size of 5 to 75 μm) and the flat under multiple environments. (H) Geckus patch grasping a 1-kg weight under vacuum, dry, moist, and underwater conditions.

To understand the adhesion characteristics of adhesives with different features, we prepared 4 samples, including 3 types of microstructures with different morphologies: (a) the Geckus structure proposed in this study, (b) the gecko-inspired mushroom-shaped adhesion microstructure (MSAM), (c) the extruded-octopus-inspired microsuckers (EOMS), and (d) a flat patch (Fig. 2A). For the convenience of comparison, the microstructured samples were all hexagonal arrays with center-to-center distances of 80 μm, the outer diameters of the structural stalks were 50 μm, and the thin-plate tips of the Geckus and mushroom-shaped structures had an outer diameter of 75 μm and a height of 30 μm. The normal adhesion of the samples to a silicon surface was measured using commercial tension machines.

To evaluate the environmental adaptability of the different samples, we measured the normal adhesion forces of the 4 samples to silicon wafers under vacuum (pressure of 5 kPa), dry (atmospheric pressure, relative humidity of 50%), moist (relative humidity of 98%), and underwater conditions. The results (Fig. 2B) show that the MSAM has excellent adhesion under vacuum and dry conditions. However, its adhesion in wet environments is reduced. This is due to the interfacial wetting and interfacial substitution of the liquid molecules present between the contact interfaces, which leads to a considerable reduction (or even a total disappearance) of van der Waals forces under moist or underwater conditions. By contrast, the adhesion of the EOMS is enhanced under wet conditions, which is due to the suction-enhanced phenomenon of the dome-like architectures and the capacity of the seal-ring tips to maintain such suction [31,32]. However, the microsuckers exhibit the lowest adhesion forces in vacuum, which is attributed to the sharp decline or even failure of suction. Under these circumstances, the van der Waals forces are the predominant forces enabling interface adhesion. In addition, the contact area of the microsuckers is limited, which results in a low adhesion.

To completely eliminate the influence of contact area in comparing the EOMS patch with the Geckus patch, we added an experiment. The EOMS structure had a terminal size of 5 to 75 μm (inner and outer diameters, respectively), matched to the Geckus structure. The normal adhesion of this EOMS patch was still weak under the vacuum conditions with 30 kPa, while the Geckus generated 120 kPa in vacuum. In addition, the normal adhesion of Geckus in other environments was significantly higher than that of the EOMS patch (Fig. S10). This clearly demonstrates that contact area is not the absolute influencing factor for adhesion strength; instead, the geometric configuration and underlying adhesion mechanisms of the structures are more critical.

It is worth noting that among the 4 environments investigated (vacuum, dry, moist, and underwater), the Geckus structure exhibits the best adhesion performance. This advantage is attributed to its unique “crack reentry” interfacial behavior: During detachment, the initial crack nucleates at the center of the contact interface, first propagates inward, and then redirects outward. This detachment mode indicates that the Geckus structure is free from edge stress concentration, thereby avoiding the rapid interfacial separation often induced by such stress concentration. Meanwhile, the crack reentry phenomenon effectively delays the detachment process and substantially increases the difficulty of detachment. Furthermore, the compressible cavity of the Geckus, together with the effective sealing provided by the inner and outer annular thin-plate tips, generates sustained suction upon stretching, further enhancing adhesion. In sharp contrast, both the MSAM under wet conditions and the EOMS in dry and wet environments exhibit edge-initiated detachment, which limits their adhesion performance. The above interfacial mechanisms will be discussed in detail in the following sections, in conjunction with experimental observations and simulation analyses.

We found that the normal adhesion of all samples (including the nonpatterned flat patch) is lower in vacuum than in a dry environment, which is consistent with previous studies [33,34]. We speculate that this phenomenon is due to 2 reasons. One is the decline or disappearance of the suction forces under vacuum, and the other is the compressibility and air permeability of PDMS. In dry environments, the adhesive forces of Geckus stem from the synergistic interplay of van der Waals forces and the suction forces. However, in vacuum environments, the suction forces drop to nearly zero due to the ultralow ambient pressure (5 kPa). Consequently, the adhesion forces are exclusively governed by the van der Waals forces arising from interfacial contact. In addition, the compressive stiffness of the structure is reduced under the vacuum conditions. As evidenced by the typical force curves of Geckus in the normal adhesion tests (Fig. 2C), the press slope is reduced in the vacuum environments compared to the curves measured in other environments, indicating a decrease in the structural compressive stiffness. This is owing to the fact that the compression of the structure needs to overcome air damping within the material under ambient pressure. Whereas in vacuum, the air within the PDMS materials is evacuated. Consequently, the structure is pressed without the air damping, manifesting as a softer material compressive stiffness. This results in a slower detachment, which ultimately affects adhesion performance.

The curves showing the changes in normal adhesion with increasing preload under vacuum, dry, moist, and underwater conditions are shown in Fig. S11. The adhesion increases for all samples with rising preload. In particular, for the Geckus structure, its compressible cavities and thin-plate tips cause a larger volumetric change and a closer interfacial contact under preload, respectively, resulting in a greater adhesion. Under vacuum conditions, where adhesion relies exclusively on van der Waals forces (which require direct contact to manifest), increased preload ensures reliable contact between the inner, outer brims of Geckus and the foreign surface. This enhances the efficiency of contact splitting and amplifies van der Waals interactions. Consequently, when preload is appropriately increased, the adhesion force rises abruptly.

To explore the effect of the thin-plate head size on normal adhesion, we manufactured samples with ring-shaped head sizes of 20 to 60, 15 to 65, 10 to 70, and 5 to 75 μm (the inner and outer diameters, respectively). The normal adhesion to a silicon surface in vacuum, dry, wet, and underwater environments was measured (Fig. 2D). We found that the adhesion forces rise with the increase in the tip size; the ring-shaped head with a size of 5 to 75 μm exhibits the maximum normal adhesion under vacuum (120 kPa), dry (201 kPa), moist (173 kPa), and underwater (165 kPa) conditions. Owing to the fact that interior protuberances induce the capillary-assisted suction, and the terminal tips serve as a pressure-sealing role; hence, the enlarged tip sizes enable superior sealing, which synergistically couples with amplified suction resulting from larger cavity volume variations to intensify negative pressure generation. This cooperative mechanism optimizes interfacial adhesion performances. Similarly, the normal adhesion of the Geckus samples with different head sizes increases with the preload, and the corresponding curves under vacuum, dry, moist, and underwater conditions are shown in Fig. S12.

Moreover, the Geckus with a head size of 5 to 75 μm maintains robust adhesion strength after 1,000 attachment–detachment cycles under vacuum, dry, moist, and underwater conditions (Fig. 2E), demonstrating remarkable stability across all tested environments.

To comprehensively characterize the adhesion performance of the Geckus structure (with a terminal size of 5 to 75 μm), we systematically evaluated its shear adhesion forces. As shown in Fig. S13, the Geckus patch exhibited remarkable shear adhesion in vacuum (204 kPa), dry (247 kPa), moist (201 kPa), and underwater (145 kPa) environments. The shear adhesion strengths in vacuum, dry, and moist environments surpassed those in the normal direction, aligning with observations from other gecko-inspired structures. However, due to the lubrication effect of the interfacial water layer, the shear adhesion under underwater conditions not only decreased compared to the normal adhesion in the same environment but also showed a significant reduction relative to dry conditions. In addition, shear detachment in vacuum environments occurred faster than in dry environments due to the absence of suction forces. Furthermore, the Geckus patch (with an area of 3 cm × 3 cm) exhibited robust peeling strength on the silicon wafer surface in 4 distinct environments: vacuum, dry, moist, and underwater conditions (Fig. S14). The adhesive force peaked during the initial near-normal detachment and subsequently decayed with peeling, ultimately achieving complete separation. Similarly, interfacial water wetting markedly impacted the separation process, resulting in lower peeling forces in moist and underwater environments compared to those under vacuum and dry conditions.

To evaluate the versatility of the Geckus adhesive on different surfaces, we further measured its normal adhesion forces on glass and chromium (metal) substrates under vacuum, dry, moist, and underwater conditions. As shown in Fig. 2F, the Geckus structure (with a tip size of 5 to 75 μm) maintained strong multienvironment adhesion performance on both tested substrates. Specifically, the normal adhesion forces measured on the glass surface were 107, 185, 161, and 153 kPa under vacuum, dry, moist, and underwater conditions, respectively; on the chromium metal surface, they were 99, 176, 152, and 147 kPa under vacuum, dry, moist, and underwater conditions, respectively.

We further examined the adhesion performance of the optimized Geckus adhesive at different temperatures. As shown in Fig. S15A, within the range of 25 to 150 °C, the normal adhesion forces on both silicon wafer and glass surfaces decrease with increasing temperature. However, even at 150 °C, the adhesion force on the silicon wafer remains as high as 178 kPa, and that on the glass remains 163 kPa. In addition, after continuous heating at 50 °C for 24 h, the adhesion forces measured at 0, 6, 12, and 24 h showed no significant degradation (Fig. S15B), indicating that the structure has good long-term stability at this temperature. These results confirm that the Geckus adhesive possesses excellent temperature stability.

To characterize the adaptability of Geckus on rough biological surfaces, we conducted adhesion measurements of the Geckus patch and flat membrane on goatskin under multiple environmental conditions (vacuum, dry, moist, and underwater). Goat skin, serving as a human skin replica, exhibits representative surface roughness. We characterized its microscale topography using confocal laser scanning microscopy, obtaining the *R*_a_ of approximately 4.3 μm and the *R*_z_ of approximately 43 μm (Fig. S16). To further investigate the nanoscale topography of the goat skin surface, we performed atomic force microscopy measurements over a scan area of 5 μm × 5 μm. As shown in Fig. S17, the surface height variation ranges from −322.8 to +317.3 nm, with a root mean square roughness (*R*_q_) of 92.7 nm. These results indicate that the goatskin surface is not only rough at the microscale but also exhibits nanoscale undulations, which reduce the actual contact area and consequently significantly weaken the van der Waals forces. Nevertheless, the Geckus exhibited considerable normal adhesion on goat skin under vacuum (26.9 kPa), dry (29.8 kPa), moist (23.7 kPa), and underwater (17.6 kPa) conditions (Fig. 2G), demonstrating superior performance relative to the flat one. The reduction in underwater adhesion strength compared with dry conditions is primarily attributed to liquid entrapment within surface valleys, which promotes interfacial wetting-induced detachment. These findings substantiate the potential of the Geckus patch for reliable epidermal adhesion applications.

To investigate the influence of scale on the adhesion to the Geckus structure, we used micro–nano 3D printing to fabricate an inverse-template architecture (Fig. S18A) with 40× geometric scaling relative to the prototype and then obtained the scaled-up Geckus patch by molding. The patch maintains consistent normal adhesion in vacuum (61 kPa), dry (86 kPa), moist (79 kPa) and underwater (74 kPa) environments even after 100 cycles (Fig. S18B). However, the adhesion forces decline compared to the initial scale of Geckus, aligning with contact splitting theory of size effects [35]. Specially, the thickness of the scaled-up Geckus rises to 200 μm, which induces inefficient contact, greatly reducing the adhesion.

The strong, reversible adhesion of the Geckus in multiple environments is demonstrated by its ability to stably lift a 1-kg weight in vacuum, dry, moist, and underwater environments (Fig. 2H and Movie S1). In practice, the mass an adhesive can lift depends on both its intrinsic adhesion strength and the actual effective contact area. Theoretically, an infinite sample area would impose no upper limit on liftable mass. For a finite area, however, unavoidable angular error reduces the actual contact area, making the actual adhesion force lower than the theoretical value. To test the load capacity of a finite-area sample under practical conditions, we used a specimen of 3 cm × 3 cm and found that it could stably lift a 2-kg mass (Fig. S19). This confirms that, despite contact loss due to angular error, a well-designed adhesive structure can achieve high load-bearing performance even with a limited contact area.

To better illustrate the outstanding performance of Geckus, we compared the multienvironment (vacuum, dry, moist, and underwater) adhesion properties of Geckus and other typical adhesion structures, such as the OIAs (octopus-inspired architectures) [31], AOC (amphibian-like patches with octopus-like convex cups) [36], μ-SC (microsuckers) [37], EOMS (extruded octopus-inspired micro-suckers) [32], EDA (electrothermal dry adhesives) [38], and DIA (diving beetle-inspired architecture) [39] (Table S4). Specifically, compared with the wet-tolerant adhesive OIAs, Geckus mainly focuses on multienvironment adhesion abilities and significantly improves the normal adhesion under dry, moist, and underwater conditions (201, 173, and 165 kPa, respectively, in our paper compared to 25, 37, and 41 kPa, respectively, reported for OIAs). This can be attributed to the efficient contact splitting method and discrete plate terminals of Geckus, while the connected tip surface of OIAs can cause incomplete contact and rapid crack propagation during the attachment and detachment process. In short, Geckus not only has more comprehensive environmental adaptability than other structures but also exhibits higher adhesion performance in each specific environment, which reflects the advancements and innovations of our work.

### Interfacial physical behaviors comparison of Geckus, EOMS, and MSAM

As shown in the above section, the Geckus structure exhibits the highest adhesion in all environments: higher than MSAM under vacuum and dry conditions and higher than the wet-tolerant EOMS in moist and underwater environments. Under dry conditions, van der Waals forces are the key interfacial adhesion forces relied upon by the 3 adhesive structures. They depend on direct, intimate solid–solid contact, and the contact separation mode strongly influences their magnitude. To reveal the underlying adhesion mechanism of the Geckus structure under dry conditions, we investigated and compared the interfacial physical behaviors of Geckus, EOMS, and MSAM on smooth glass during detachment using in situ optical microscopy (Fig. S20).

**Fig. 3.**
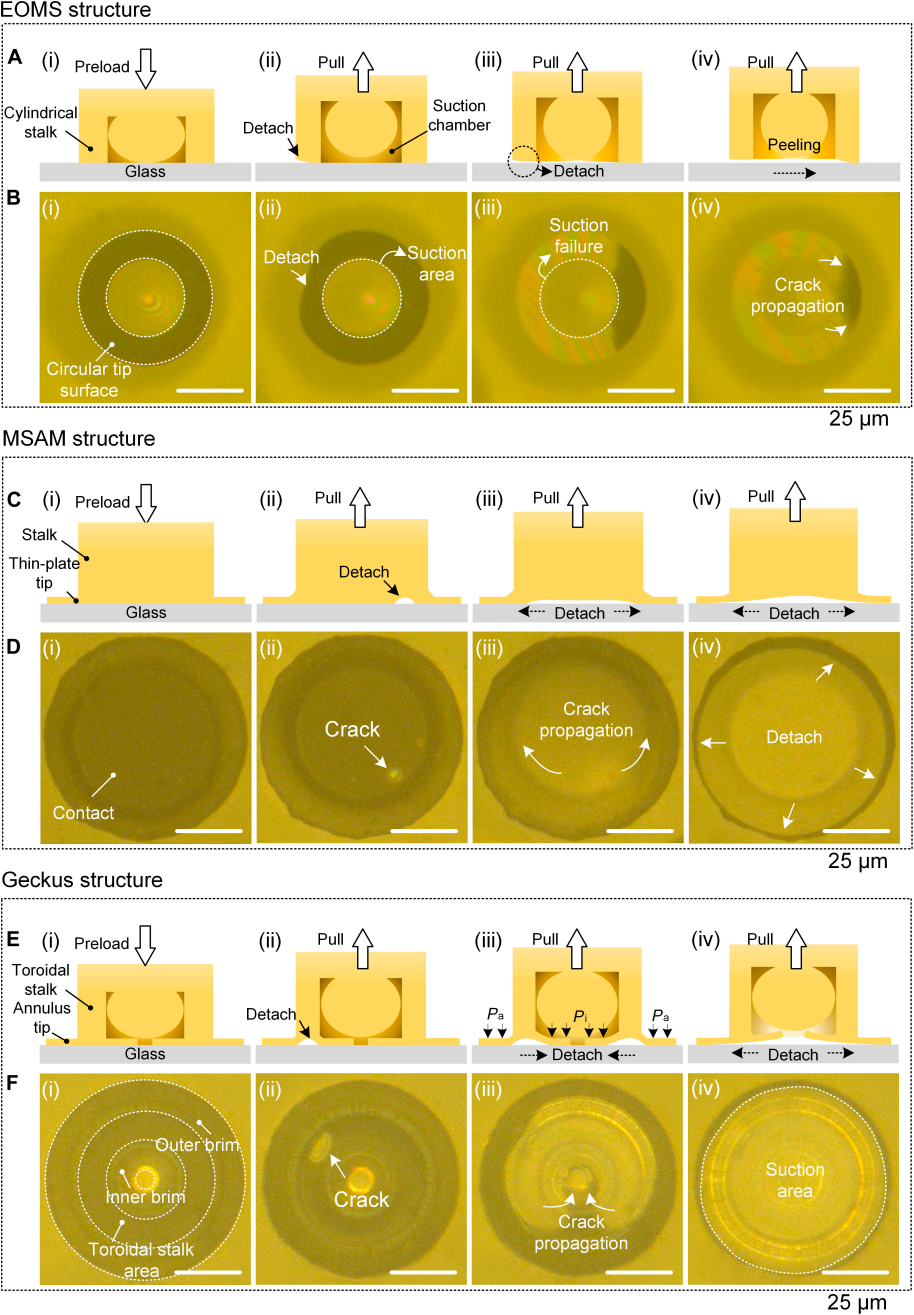
Detachment of the extruded-octopus-inspired microsucker (EOMS), mushroom-shaped adhesion microstructure (MSAM), and Geckus structures from a glass surface in a dry environment. (A) Schematics and (B) optical images of the detachment of the EOMS: (i) initial state under a preload; (ii) interfacial detachment starts at the edge of the structure; (iii) the suction effect of the central chamber vanishes as interfacial detachment occurs; (iv) the cracks continue to spread rapidly from the structural side to another side. (C) Schematics and (D) optical images of the detachment process of the MSAM structure: (i) the structure is under a preload; (ii) cracks are nucleated in the central area of the structure; (iii) cracks spread outward from the interface; (iv) the cracks continue to spread until the detachment. (E) Schematics and (F) optical images of the detachment process of the Geckus structure: (i) Geckus structure under a preload; (ii) cracks are nucleated in the central stalk area at the start of the detachment process; (iii) cracks spread along the inner brim; (iv) the inner brim is totally detached, and the cracks spread along the outer brim.

The circular tip surface of the EOMS is in close contact with the glass surface during attachment [Fig. 3A(i), under a preload of 50 kPa]. Interfacial detachment initiates at the edge of the cylindrical stalk and proceeds along the circular tip surface as the EOMS stretches until it is completely detached from the glass surface [Fig. 3A(ii) to (iv) and Movie S2]. Crack initiation at the edge of the contact interface is caused by the considerable stress concentration at the edge of the structure due to the stalk–plane contact (Fig. S21), which leads to faster separation and lower interfacial van der Waals force [40]. Significantly, the negative pressure in the central chamber is destroyed as long as the cracks propagate to the inner edge of the cylindrical stalk. To be specific, detachment at the edge of the inner circle causes damage to the sealing due to the fact that the negative-pressure chamber is connected to the outside atmosphere. It is evident that cracks are easily formed and the negative-pressure state is easily destroyed for this type of interface mechanical behavior, thus the van der Waals forces and the negative pressures are both limited. The adhesion of the artificial microsuckers under dry conditions is mainly enabled by van der Waals and suction forces. Thus the desorption threshold is moderate.

We observed the contact splitting behaviors of MSAM in dry environments (Fig. 3C and D and Movie S3). In contrast, crack initiation occurs within the interface [Fig. 3D(ii)]. As the pulling process progresses, the crack propagates radially outward from the initiation point until complete detachment. This T-shaped configuration, specifically, the integration of a thin plate tip at the terminal of the supporting stalk, has been demonstrated to significantly improve the stress concentration at structural edges, thereby effectively inhibiting crack propagation. This mechanism fundamentally contributes to the superior adhesive performances of MSAM in dry environments.

The Geckus consisting of the stalk and the thin-plate tip inherits the configurational advantages of the T-shape of the MSAM, which can efficiently prevent crack propagation [41]. As shown in Fig. 3F(i), we define the inner annulus of the thin-plate tip as the inner brim and the outer one as the outer brim. During the detachment of the Geckus from the glass surface under dry conditions (Movie S4), cracks are initiated at the middle of the contact interface rather than at the edge [Fig. 3F(ii)]. This is due to the homogeneous interfacial stress distribution in the plate–plane contact mode, which renders crack generation more difficult than in the stalk–plane contact mode (Fig. S21). As shown in Fig. 3F(iii), once nucleated, the cracks gradually spread toward the inner brim. This is due to the different pressures that the inner and outer brims are subjected to. The inner sealed chamber (*P*_i_) and the outside atmosphere exert a pressure (*P*_a_) on the inner and outer brims, respectively. During the detachment process, the inner chamber is in a negative-pressure state due to the tensile deformation of the Geckus; this negative pressure is lower than the external atmospheric pressure, and, thus, the cracks can easily propagate to the inner brim.

Remarkably, the negative-pressure state in the central chamber is not destroyed even when the inner brim and the foreign surface are fully detached, which is due to the fact that the outer brim is still fixed to the glass and thus maintains the sealing. In this case, the action area of negative pressure becomes larger. To be precise, the suction area expands to comprise the regions enclosed by the inner brim and the toroidal stalk surface, while the intermolecular forces are reduced because of the smaller contact area. Upon increasing the structural deformation during detachment, the cracks spread again from the center to the outer brim, which is the opposite direction compared with the initial propagation. We define this phenomenon as the crack reentry behavior. The suction effect vanishes once the outer brim is detached from the glass.

From the above observations, the T-shaped configuration of Geckus and MSAM causes cracks to initiate from the center with a flat stress distribution, making detachment more difficult than for EOMS, which initiates from the edge, thus generating larger interfacial adhesion. In wet environments, however, liquid replaces the solid–solid interface, causing van der Waals forces to greatly weaken or even disappear.

**Fig. 4.**
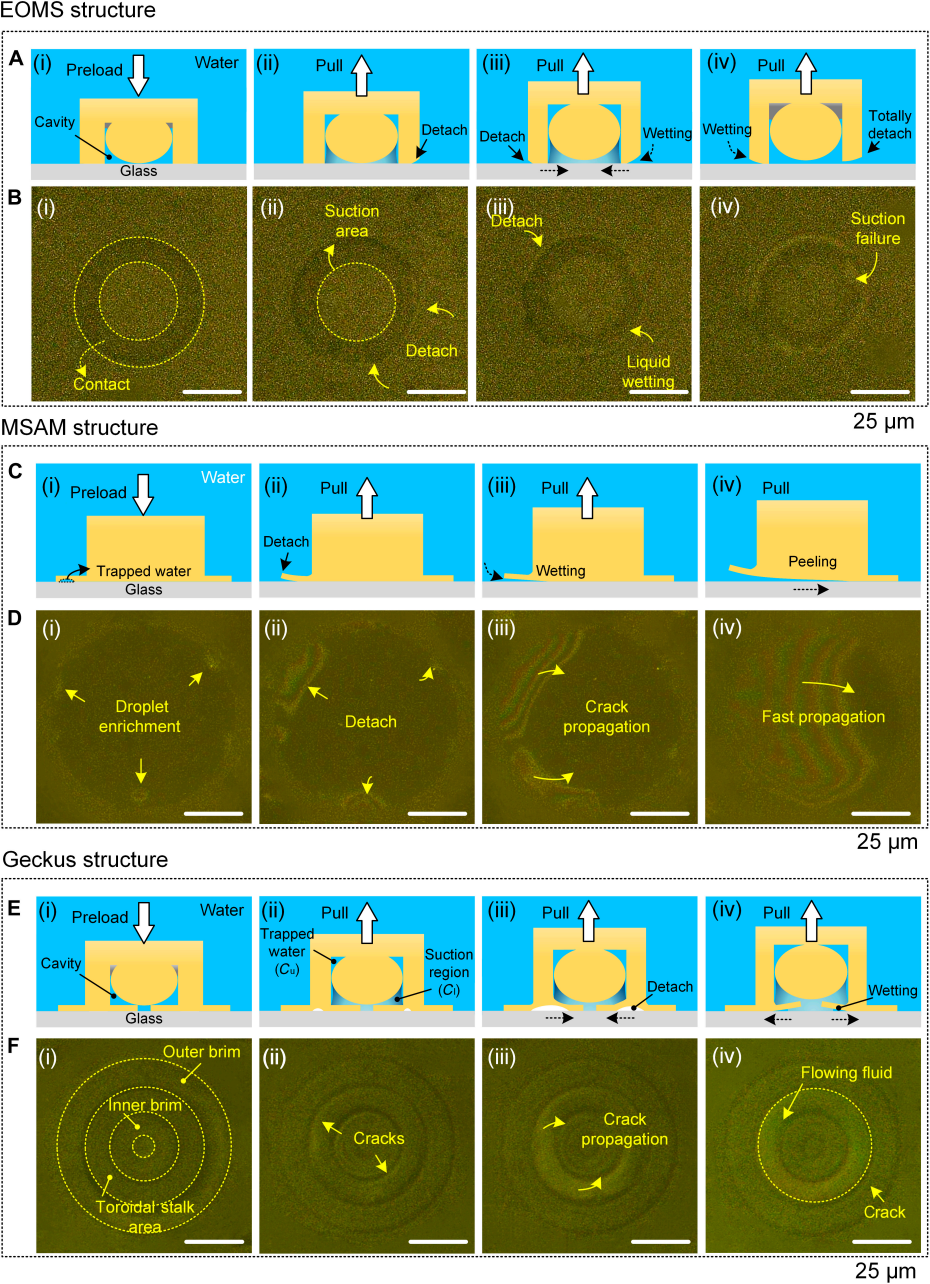
Detachment of the mushroom-shaped adhesion microstructure (MSAM), extruded-octopus-inspired microsucker (EOMS), and Geckus structures from a glass surface in a wet environment. (A) Schematics and (B) optical images of the detachment process of the MSAM structure: (i) initial state under a preload; (ii) several cracks develop at the edge of the MSAM tip; (iii) the cracks continue to propagate upon pulling; (iv) the cracks converge together and propagate quickly. (C) Schematics and (D) optical images of the detachment process of the EOMS structure: (i) initial state of EOMS under preload; (ii) the initial cracks are generated at the structural edge under the pulling of the structure; (iii) cracks spread inward under the wetting of liquid; (iv) the failure of suction owing to the destruction of sealing. (E) Schematics and (F) optical images of the detachment process of the Geckus structure: (i) initial state of the Geckus structure under a preload; (ii) the initial cracks are generated at the center of the contact interface owing to the pulling of the structure; (iii) cracks develop along the inner brim of the structure; (iv) the inner brim detaches from the foreign surface, a small amount of fluid flows around the detached area, and the cracks spread along the outer brim.

To understand the possible reasons for the strong adhesion of the Geckus in a wet environment, we explored and compared the different interfacial physical behaviors of MSAM, EOMS, and Geckus upon detachment from a flat glass surface in an underwater environment. According to the previous performance characterization, the MSAM is inefficient underwater. Figure 4A(i) shows the initial state of MSAM under a preload of 50 kPa. It can be observed that there is some liquid between the MSAM tip and the glass surface. Figure 4A(ii) shows the state of MSAM at the beginning of the detachment process. It can be seen that the edge of the circular plate tip is the first to detach. The cracks then continue to propagate along the previous ones, and eventually the circular plate tip detaches effortlessly from the glass surface [Fig. 4A(iii) and (iv)] upon further pulling. We found that the circular plate tip and the foreign surface were separated by a thin water film owing to the interface wetting of the liquid (Movie S5). Since the MSAM is nonporous and has a flat tip surface, it is prone to undergo droplet enrichment, which causes the generation of precracks at the contact interface. This situation could result in the MSAM losing its structural advantages, that is, the fact that the cracks nucleate at the center of the interface under tight contact in a dry environment. Moreover, the precracks are generated at several places on the contact interface, especially at the edge of the thin plate; these cracks are more likely to cause interfacial splitting. Therefore, the detachment process starts easily from the edge of the thin-plate head and proceeds rapidly due to interface wetting and division by the liquid molecules that accumulate at the detached critical area, resulting in the limitation of adhesion ability under wet conditions.

The EOMS has a cavity with a microdome, which is capable of generating capillary-assisted suction. However, similar to MSAM, it still exhibits edge-initiated detachment (Movie S6). As illustrated in Fig. 4C and D, when cracks nucleate at structural edges, they progressively propagate inward because of liquid wetting effects until reaching the boundary of the suction area. This progression causes seal failure and consequent dissipation of suction forces. Distinct from MSAM’s T-shaped configuration, the EOMS fundamentally consists of a perforated stalk integrated with a microdome in the cavity. During contact, unavoidable stress concentration at structural edges leads to detachments from the edges. This explains why the EOMS has the underwater adhesion capability but still remains less efficient.

The Geckus contains cavities, which can store the superfluous, squeezed liquid at the interface and can also be compressed and stretched to generate volume changes and drive suction. In the preload process, the liquid between the Geckus tip and the foreign surface is squeezed out of the contact area or drained into the central cavity. It should be emphasized that the structural swelling phenomenon does not occur during this process. This is due to the material used, PDMS, which is superhydrophobic and exhibits an exceptionally low swelling ratio in water; hence, it can fundamentally prevent water infiltration into the material and avoid the structural swelling. It was observed that no liquid enrichment occurred between the Geckus tip and the glass surface after a 50-kPa preload [Fig. 4F(i)]. Similar to the detachment process under dry conditions, the interfacial cracks still nucleate from the toroidal stalk area under the action of pulling-off forces under underwater conditions, as shown in Fig. 4F(ii) and Movie S7. Due to the incompressibility of the liquid, most liquid is sucked into the upper chamber (*C*_u_) of the central cavity that is divided by the microdome [31], and then a capillary-assisted suction region is formed in the lower chamber (*C*_l_) due to the recovery and deformation of the Geckus structure. It is worth noting that the crack reentry phenomenon also occurs in wet environments during contact splitting. The formed cracks first propagate along the inner brim upon further pulling, as shown in Fig. 4F(iii). Liquid molecules that exist in the areas of the toroidal stalk and the inner brim are sufficient in number to aggregate together and flow around the interface after the complete detachment of the inner brim, as shown in Fig. 4F(iv). Finally, the cracks spread along the outer brim until complete detachment from the glass surface occurs because of the wetting of the flowing liquid.

The above crack nucleation and propagation patterns were consistently observed across multiple independent experiments. To further verify the reproducibility of these observations, we performed additional in situ observations of underwater interfacial detachment on the 3 structures (MSAM, EOMS, and Geckus), all of which exhibited the same crack initiation and propagation behavior (Fig. S22), as shown in Fig. 4. This fully validates the reliability of the detachment phenomenon observed in this work and the proposed mechanism.

In summary, Geckus exhibits the highest adhesion in diverse environments, owing to 3 synergistic characteristics: (a) crack reentry behavior, where cracks nucleate at the center of the interface and first propagate inward and then outward, effectively suppressing crack separation; (b) a compressible central cavity with a microdome that generates sustained suction under tension and can also store compressed liquid in wet environments to maintain intimate contact; and (c) dual sealing provided by the inner and outer annular thin-plate tips, preserving suction even after the inner edge detaches. In contrast, although the MSAM possesses structural advantages, which prevent crack propagation under dry conditions due to its T-shape construction, the fact that the tip has a nonporous surface means that such advantages do not hold under wet conditions; this is due to the interfacial liquid enrichment phenomenon, which affects the tightness of the interfacial contact. Although EOMS has a cavity, its stalk–plane contact mode results in excessively small van der Waals forces under dry conditions, causing negative pressure to fail quickly. By contrast, through the combination of the thin toroidal-shaped plate tip and the central cavity with the dome-like structure, the Geckus structure does not exhibit interfacial stress concentration, so that it forms the unique crack reentry interface physics and inhibits crack propagation. Thus, whether under dry or wet conditions, the cavity with the dome-like structure and the thin toroidal-shaped plate tip of the Geckus are indispensable elements, which promote adhesion in multiple environments by inhibiting crack propagation, increasing negative pressure, and providing a tight seal.

### Adhesion mechanisms of the Geckus under dry/wet conditions

**Fig. 5.**
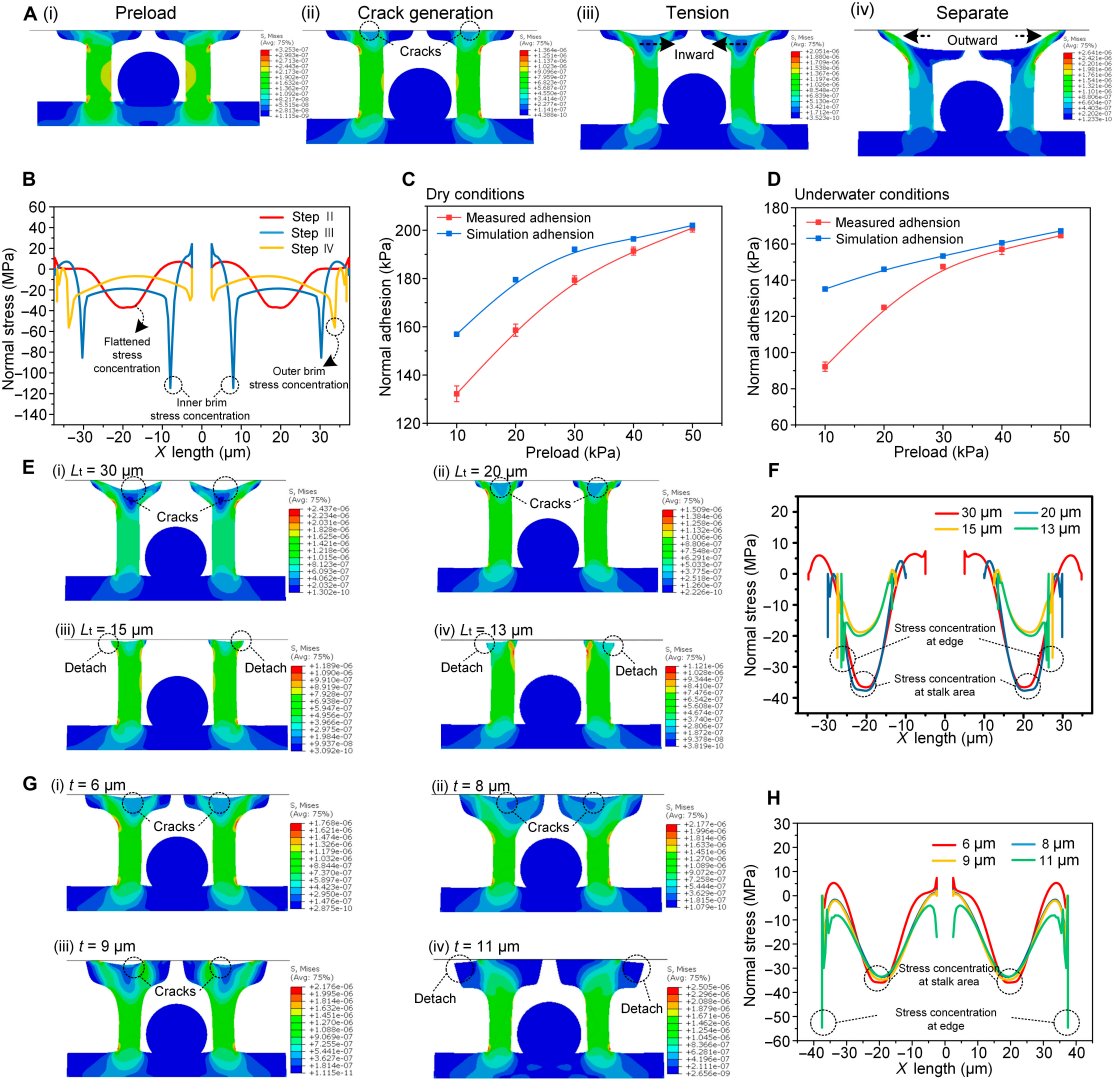
The adhesion mechanism of Geckus and the conditions of crack nucleation. (A) The stress distribution states of Geckus in the contact separation numerical analysis (dry environments). (B) The normal stress distribution of contact interface at the moments of crack generation, tension and separation, respectively. The comparisons of predicted and measured normal adhesion of Geckus structure (tip size of 5 to 75 μm) under both (C) dry and (D) underwater conditions. (E) Stress distribution states of Geckus structures with different tip lengths (*L*_t_ = 30, 20, 15, and 13 μm, respectively) at the moment of crack generations. (F) Interfacial distribution of normal stress contained Geckus structures with varied parameter *L*_t_ (30, 20, 15, and 13 μm) at the moments of crack nucleation. (G) Stress distribution states of Geckus structures with different tip thicknesses (*t* = 6, 8, 9, and 11 μm, respectively) at the moment of crack generations. (H) Interfacial distribution of normal stress of Geckus structures with varied parameter *t* (6, 8, 9, and 11 μm) at the moments of crack nucleation.

To further clarify the adhesion mechanisms of Geckus, we established theoretical contact separation models of Geckus under both dry/wet conditions. Figure 5A illustrates the preload–detach process of Geckus under dry conditions. The initial cracks nucleate from the annular stalk area, then spread inward, and eventually redirect outward. This behavior is consistent with the actual interface behaviors we captured. The analysis reveals that the Geckus structure maintains a relatively flat interfacial stress distribution without notable stress concentration during initial contact (Fig. 5B). Stress concentrations emerge at the edges of the toroidal stalk area under sustained pulling, triggering crack initiation at this area. The inner brim region exhibits more pronounced stress concentration compared to the outer brim region as pulling continues. This is the reason why the cracks consistently propagate inward first.

In dry environments, the normal adhesion forces of Geckus arise from both van der Waals forces and suction. During structural compression and subsequent tensile deformation, the volume change of the Geckus cavity generates a negative pressure within the cavity. Since the external pressure remains at atmospheric pressure, the resulting pressure differential induces suction. As long as the interface remains intact, greater structural deformation leads to stronger suction under sustained pulling. Meanwhile, considerable van der Waals forces are generated through the unique interfacial behavior of Geckus, which preserves reliable interfacial contact and ensures effective sealing for strong suction. To distinguish the contributions of these 2 components, we compared the normal adhesion measured under dry and vacuum conditions (Fig. S23A). Under vacuum conditions, suction forces become zero, leaving only van der Waals forces. Therefore, the difference between the dry and vacuum adhesion values represents the suction force under dry conditions. Both van der Waals forces and suction forces increase with enlarging tip size.

In contrast to dry conditions, the normal adhesion forces in underwater environments consist of capillary forces and suction. Here, capillary forces generated by capillary bridges replace van der Waals forces to ensure interfacial bonding and contact. Our model quantifies the contributions of capillary forces and suction to the total normal adhesion (Fig. S23B), revealing that they both increase with tip size. Clearly, suction force depends on both the acting area and the pressure differential. Although a larger tip has a smaller orifice for the initial suction acting area, it can generate stronger capillary forces. These stronger capillary forces, in turn, amplify the cavity pressure differential, namely achieve greater deformation through strong sealing, and thereby enhance the total adhesion force.

The model can accurately predict the normal adhesion of Geckus (tip size, 5 to 75 μm) under preload pressures ranging from 10 to 50 kPa in both dry and underwater environments (Fig. 5C and D). However, at low preload pressures (e.g., 10 kPa), a certain discrepancy is observed between the measured adhesion and the simulation results. This is mainly attributed to the deviation between the actual contact state and the ideal model assumption. Under extremely low preload pressures, the actual contact interface may not achieve tight contact due to minor angular error, whereas the simulation assumes an ideally perfect contact state at the interface. Consequently, the measured adhesion is lower than the simulated predictions. As the preload pressure increases, the contact interface becomes more sufficient, and the actual contact state approaches the model assumption, leading to good agreement between the experimental values and the simulation results.

The results of interfacial behaviors, simulation analyses and experimental data indicate that the ways of crack nucleation are critical. Studies have shown that the tip diameter and thickness are important structural geometric features that influence crack nucleation [40,42]. To further explore the influence of structural geometric features on crack nucleation, we conducted contact splitting simulations considering the variable tip sizes and tip thicknesses of Geckus. Here, we define the width of Geckus stalk as parameter *L*_s_, the width of the terminal annular tip as parameter *L*_t_, and the thickness of the tip as parameter *t* (illustrated in Note S2).

For the influence of different tip sizes, we fixed other structural parameters (height = 30 μm, chamber width = 30 μm, *L*_s_ = 10 μm, and *t* = 5 μm) and varied the terminal annular tip width *L*_t_ from 13 to 30 μm. We found that when *L*_t_ was greater than 15 μm in this set of comparative models, the cracks tended to generate at the interface region of the toroidal stalk [Fig. 5E(i) and (ii)]; this is because stress concentration always occurred in this area before the critical point of crack initiation (Fig. 5F). When *L*_t_ fell to 15 or 13 μm, the structure detached from the tip edge [Fig. 5E(iii) and (iv)]. This is because prominent stress concentration occurred at the structural tip edge (Fig. 5F), subsequently leading to rapid detachment.

As for the influence of thickness on crack nucleation, we fixed the structural height at 30 μm, chamber width at 30 μm, *L*_s_ at 10 μm, and *L*_t_ at 35 μm and varied tip thickness *t* from 6 to 11 μm [Fig. 5G(i) to (iv)]. We observed that when thickness increased to 11 μm, cracks transitioned from nucleating at the toroidal stalk region to initiating at the structural edge [Fig. 5G(iv)]. By analyzing the interfacial stress distribution states prior to the critical point of crack initiation (Fig. 5H), it was found that as thickness increased, interfacial stress shifted from concentrating at the toroidal stalk region to becoming sharply concentrated at the structural edges. This indicates that increasing tip thickness diminishes the structural advantages of the Geckus. To further quantitatively establish the relationship between the crack reentry behavior and adhesion enhancement, we compared the adhesion forces of 2 Geckus models with tip thicknesses of 9 and 11 μm, respectively, under the same preload. The results show that under the same preload, the normal adhesion force of the structure with the crack reentry effect (*t* = 9 μm) is significantly higher than that of the structure without this effect (*t* = 11 μm) (Fig. S24), which directly confirms that the crack reentry behavior plays a important role in enhancing adhesion performance.

### Application of the Geckus patch to multienvironment epidermal electronics

To demonstrate the multienvironment adhesion ability of Geckus patch, we developed a skin-attachable electronic device integrating a reliable interfacial adhesion layer and a strain-induced crack sensor. In wearable electronics, maintaining a conformal adhesion to the human skin is crucial but challenging. It is difficult for a patch to tightly and seamlessly adhere to the rough skin epidermis while following human motion. Especially, when there exists moisture on the skin, devices are more likely to detach under the impact of a liquid barrier between the skin and the device. For applications to the aerospace and high-altitude situations, the adhesion ability at low pressures should also be considered. The proposed multienvironment Geckus adhesive can efficiently adapt to the abovementioned application scenarios, and it can thus provide a reliable alternative for the attachment of wearable electronic devices in multiple environments.

The Geckus structure proposed in this work features a microdome within its hollow cavity, and its wet adhesion mechanism relies on the synergistic action of capillary and suction forces. This design enables tolerance to salt-containing sweat and sebum. On one hand, the cavity accommodates excess interfacial sweat, thereby reducing the liquid layer thickness at the contact interface, which can enhance capillary force and ensure reliable adhesion. Similar strategies have been experimentally confirmed to improve wet adhesion in earlier studies [22]. On the other hand, the high viscosity of oils promotes effective suction sealing, which can strengthen adhesion. Therefore, this structure is well suited for real skin environments. In addition, this adhesive patch also has excellent biocompatibility by applying the PDMS material [43].

To evaluate the reliability of the device under real-world moist skin-attachment scenarios, we tested the peeling force of Geckus on moist goat skin. The tests were conducted under both ambient and low-pressure (30 kPa) conditions. The results show that the maximum peeling force is 0.53 N under ambient pressure and 0.44 N under low pressure on moist goat skin (Fig. S25). These data indicate that the Geckus adhesive possesses good interfacial adhesion ability for actual skin-attachment use, serving as a reliable interfacial adhesion strategy for skin-attachable electronic devices under diverse environmental conditions.

**Fig. 6.**
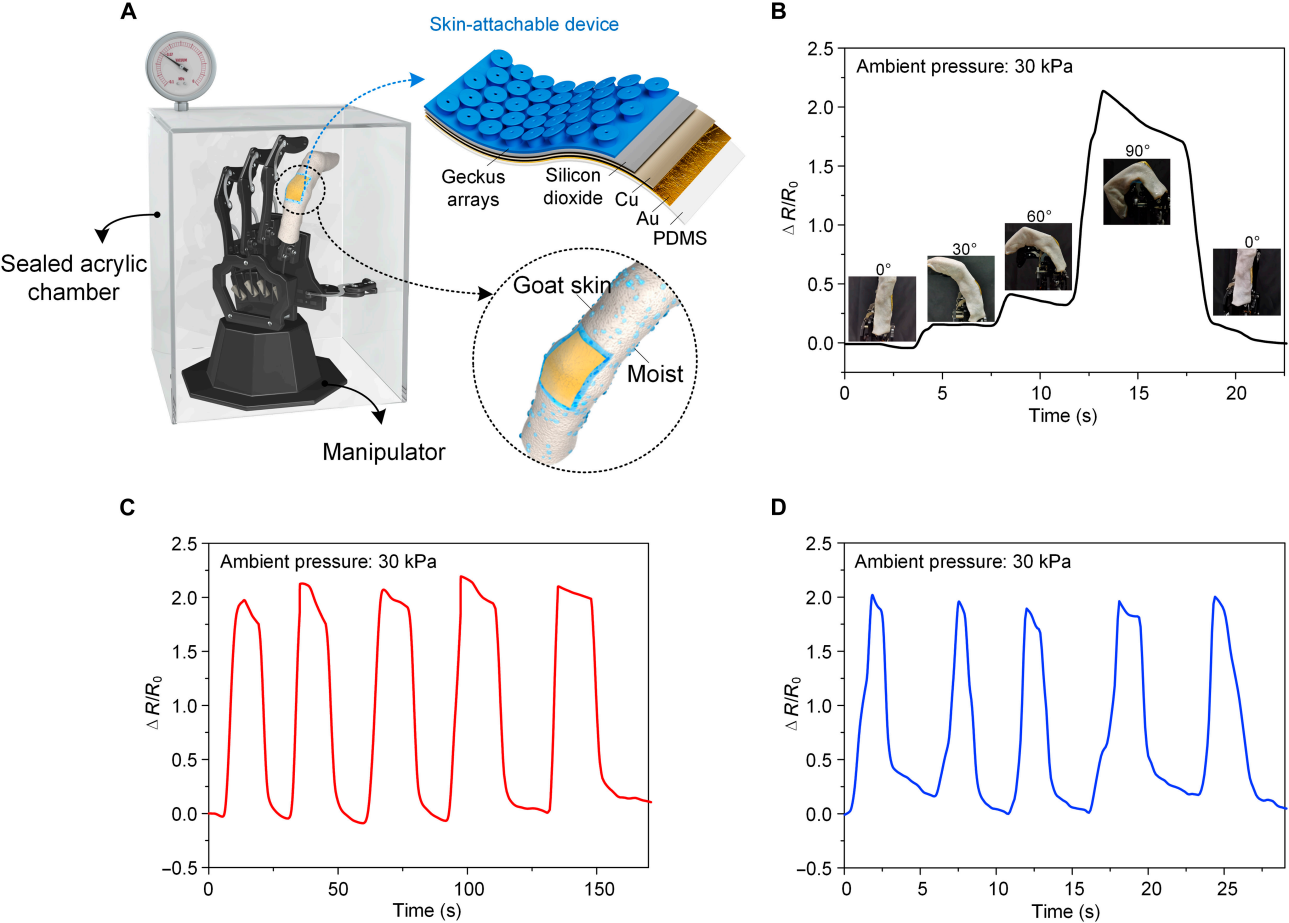
Application of the Geckus patch to an epidermal electronic device on a wet skin replica under low-pressure conditions. (A) Schematic of the proposed device applied to a goat skin-wrapped manipulator in a sealed acrylic chamber; the insets are the magnified view and diagram of the structure of the device. (B) Follow-up response of the device at different bending angles of the manipulator finger. Resistance changes with time as the wet manipulator finger bends at (C) slower and (D) higher frequencies in a low-pressure environment.

Here, the Geckus patch was used as the adhesion layer of a skin-attachable electronic device to achieve efficient and conformal adhesion on a skin replica or human skin (Fig. 6A). The sensor part consisted of thin metal layers, including Cu and Au, which can produce resistance variation signals under the introduction of metal cracks when the device is deformed under tension or bending. The adhesion layer and metal sensor layers were integrated via an ultrathin SiO_2_ layer, and the device was finally packaged using PDMS. The overall size of the skin-attachable electronic device was 1 cm by 2.5 cm, and its thickness was 200 μm. This device was used for monitoring in real-time finger bending motions.

To more comprehensively evaluate the sensing performance of this wearable electronics, Fig. S26A shows the relationship curve between relative resistance change and strain within the strain range of 0% to 5%. As the strain increases, the resistance change of the device also increases. We fitted the slope of this curve and obtained a gauge factor of 157. The device was fixed on a lead screw, stretched at a speed of 2 mm/s, and then returned to its initial state at the same speed. The response time and recovery time were observed to be 130 and 210 ms (Fig. S26B), respectively. These data indicate that the device exhibits good dynamic performance and can generate sensitive following responses.

The pressure inside a spacesuit is usually maintained at around 30 kPa to avoid decompression sickness [44–46], while the pressure in a plateau environment is often higher than this value. To simulate the actual operation environment, we measured the sensing performance of the skin-attachable device in a sealed acrylic box at a pressure of 30 kPa. Here, a manipulator was placed within the acrylic box and remotely controlled to simulate the bending movements of the finger joint of a human hand (Fig. S27). The index finger of the manipulator was wrapped with the goat skin, which was used as a human skin replica since its epidermal morphology and skin thickness are similar to those of the human skin and it has a good stretchability [47]. We found that the device was tightly and conformally attached to the rough skin replica (Fig. S28A) and was capable of deforming with the bending of the finger even when the skin surface was moist in a low-pressure environment. Based on this, the device was capable of capturing the bending of the manipulator finger joint, which varied from 0° to 30°, 60°, and 90°, providing a fast response (in terms of the resistance change) and a good recovery (Fig. 6B). In addition, the device could provide a high-sensitivity and high-resolution response under multiple bending cycles from 0° to 90° at different frequencies (Fig. 6C and D).

**Fig. 7.**
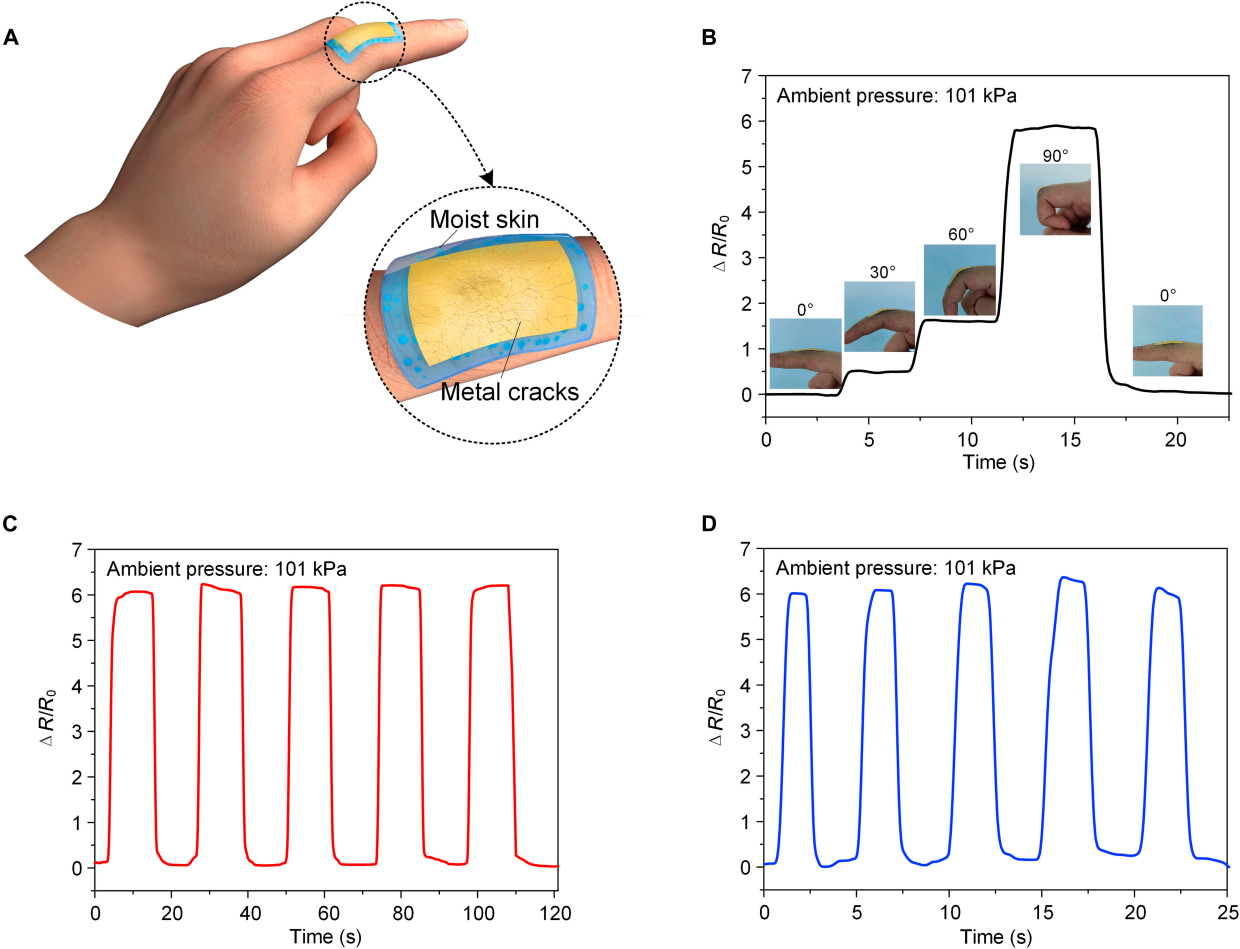
Application of the Geckus patch to an epidermal electronic device on wet human skin under atmospheric dry conditions. (A) Schematic of the proposed device applied to a human finger; the inset shows the magnified view of the patch to illustrate the wet skin surface and the metal cracks of the skin-attachable device under stretching. (B) Follow-up response of the skin-attachable device at different bending angles of the finger. Resistance changes with time as the wet human finger bends at (C) slower and (D) higher frequencies in a dry environment.

Similarly, we characterized the properties of this device attached to wet human skin under atmospheric conditions (Fig. 7A and Fig. S28B). The device can tightly adhere to and follow the deformation of the finger skin, so that it can monitor the bending motion of the finger joint and generate corresponding resistance changes (Fig. 7B). It is worth noting that the resistance remains relatively stable during bending in Fig. 7B, whereas in Fig. 6B, the resistance exhibits a trend of first increasing and then slightly decreasing over the bending cycles. This difference mainly stems from the different bending actuation methods used in the 2 experiments. In the test of Fig. 6B, the bending was performed by a robotic hand wrapped with goat skin to simulate a human hand under low-pressure, moist conditions. The robotic hand is driven by a motor; when bending to a set angle, the sudden stop of the motor generates inertial impact and mechanical backlash, causing the sensor to undergo a momentary strain surge, after which it returns to a relatively stable position. This “overshoot-recovery” process leads to the initial increase followed by a slight decrease in resistance. In contrast, in the test of Fig. 7B, bending was performed on a real human hand, which is controlled by muscles and moves smoothly and precisely without mechanical impact or backlash. Consequently, the sensor undergoes stable and sustained strain, and the resistance shows a relatively constant plateau during the bending–holding period. Furthermore, it still has good repeatability and stable responses under the slow and fast bending cycles with bending angles ranging from 0° to 90° (Fig. 7C and D).

In addition, we attached the proposed wearable electronic device to moist human finger skin for 12 consecutive hours. The resistance change of the sensor was measured at 0, 6, and 12 h during finger joint bending from 0° to 30°, 60°, 90°, and back to 0° (Fig. S29). The response curves obtained from the 3 measurements showed excellent consistency, with virtually no deviation. These results indicate that the Geckus adhesive can maintain firm adhesion to human skin over extended periods, demonstrating good operational stability and thus supporting its long-term application in wearable electronics.

To comprehensively evaluate the stability and reliability of the device in practical application environments, we conducted multiple adhesion/detachment cycle tests and bending cycle tests. The results show that under 4 typical environments (vacuum, dry, humid, and underwater), the adhesion force of the device on the goat skin surface did not show substantial degradation after 100 repeated attachment/detachment cycles (Fig. S30), indicating that the device possesses good environmental adaptability and reusability. The bending cycle test results show that after 1,000 repeated bending deformations, the resistance change rate of the device remained consistently around 2.7 (Fig. S31), further confirming that the sensor can maintain an almost unchanged resistance response under repeated mechanical deformation. These experimental results demonstrate that the proposed attachable electronic device not only exhibits excellent adhesion stability but also outstanding resistance to bending fatigue, meeting the stability and reliability requirements of skin-wearable devices in practical application scenarios.

Hence, the proposed Geckus patch can stably and reliably attach to wet skin in atmospheric-pressure and even low-pressure environments due to its strong adhesion in multiple environments, which provides potential applications in wearable devices for complex conditions, such as sweaty skin surfaces, or extreme environments, including space and plateau.

## Conclusion

In conclusion, this paper presents a novel technology for the reversible and reliable attachment of wearable electronics in complex and dynamic environments. Through dual biomimetic approaches, a new adhesive microstructure named Geckus is designed. It can overcome the inherent limitations and environmental conflicts of conventional adhesion methods across vacuum, dry, moist, and underwater conditions. Distinct from our previously reported hollow-mushroom-shaped adhesive [48], which relies solely on intermolecular forces and suction due to the absence of a microdome, the present Geckus adopts a microdome architecture inspired by the octopus sucker. This introduces a capillary-assisted effect and forms a coupled mechanical mechanism of van der Waals force, suction, and capillary force. Notably, we report for the first time a unique crack reentry contact splitting phenomenon in this structure. Specifically, cracks originate from the stalk area, propagate toward the inner rim of the structural tip, and then repropagate outward. This unusual propagation mode, enabled by the plate–plane contact configuration and the differential forces on the inner and outer brims induced by negative pressure in the hollow chamber, effectively suppresses crack growth and enhances the detachment threshold. In contrast, conventional adhesive structures (e.g., mushroom-shaped adhesives under underwater conditions or extruded microsuckers) typically detach from the contact edge, leading to faster failure and lower adhesion. The Geckus configuration inherits the advantages of both biostructures while avoiding their shortcomings, exhibiting the novel crack reentry phenomenon in both dry and wet environments, which is the main reason for the unparalleled multienvironment adhesion performances. Further simulation analyses confirm that this distinctive contact splitting manner plays a critical role in achieving robust interfacial adhesion across diverse environments by suppressing crack propagation, increasing the detachment threshold, and sustaining strong adhesion.

To fabricate this complex 3D architecture, which features an embedded microdome, a hollow cavity, and a thin annular plate tip, the conventional photolithography used in our previous study is insufficient. We therefore develop a microtemplate imprinting and backside exposure strategy, achieving large-area (3.1 cm × 3.1 cm), high-precision, and controllable molding of Geckus arrays. This technology not only offers new methods and ideas for the manufacturing of complex morphology microstructures but also holds promise for application in roll-to-roll imprinting processes to enable the fabrication of such structures over larger areas. However, roll-to-roll processing requires careful exploration of process parameters such as imprinting pressure and rotation speed, and the fabrication of these structures also necessitates supporting process technologies and equipment, including large-area photolithography and development following the imprinting. This represents an important direction for our future research.

The fabricated Geckus adhesive exhibits outstanding normal adhesion strengths in vacuum (120 kPa), dry (201 kPa), moist (173 kPa), and underwater (165 kPa) environments, as well as superior shear adhesion strengths in vacuum (204 kPa), dry (247 kPa), moist (201 kPa), and underwater (145 kPa) environments. Moreover, it demonstrates excellent adaptability to rough and uneven skin surfaces across diverse environmental conditions. A prototype ultrathin, lightweight wearable device was developed using the Geckus patch as the adhesive interface, successfully demonstrating its applicability in low-pressure wearable systems. The device adheres firmly to moist skin and maintains functionality under both low-pressure and atmospheric conditions, exhibiting sensitive responsiveness. These results validate the Geckus patch as a robust, versatile interfacial adhesive for integrating sensor components into advanced wearable systems. This work opens up promising avenues for the development of wearable electronics operating in extreme low-pressure environments, such as high-altitude or space applications.

## Methods

### Materials

The transparent glasses were purchased from Luoyang Guluo Glass Co. Ltd. (China); they were sputtered with a 10-nm-thick Cr layer and used as substrates. The photoresist (AZ P4620) was obtained from Suzhou Research Materials Microtech Co. Ltd. The flexible template and the adhesive patch were made of PDMS (Dow Corning Sylgard 184), which was purchased from Dow Corning Inc. (USA). The Si wafer was obtained from Suzhou Research Material Co. Ltd.

### Measurement and characterization of the adhesion properties

The normal adhesion data were collected via load–pull method. Specifically, a probe (with an area of 3 mm × 3 mm) was fixed on the pull rod of the tension machine, and the sample was fixed on the adjustable platform below the probe. The probe moved downward at a speed of 1 mm/min until it came into contact with the sample and reached the set preload. After applying the preload to the sample for 5 s, the probe was moved upward at a speed of 1 mm/min until it detached from the sample. The normal adhesion force corresponds to the maximum tensile force experienced by the probe during detachment. Each measurement was repeated 3 times, and the average value of these measurements was taken as the normal adhesion corresponding to the specific preload. The sample was adjusted to be parallel to the probe before the measurements. The measurements of the shear forces were achieved through the tangential movement of 2 parallel cantilever beams that are vertically fixed at the top and bottom of the tensile machine. The silicon wafer was fixed on the inner side of one cantilever beam, and the test sample (with an area of 5 mm × 5 mm) was fixed on the inner side of the other cantilever beam. Before the test, a pressure of 20 kPa was applied in the normal direction of the structure to ensure complete contact between the structure surface and the silicon wafer. The cantilever beam with the silicon wafer was pulled upward at a speed of 5 mm/min until the silicon wafer was completely separated from the test sample. The test of the peeling force was carried out by fixing the silicon wafer on the test bench, applying a preload of 20 kPa to ensure that the adhesion sample (with an area of 3 cm × 3 cm) was fully adhered to the surface of the silicon wafer, and then pulling the adhesion sample upward at a speed of 60 mm/min until the sample was completely peeled off from the silicon substrate. The adhesion forces under vacuum (5 kPa) were measured using a vacuum tension machine (PT-1176, Baoda, China). The adhesion forces in dry environments were measured using a computer servo pull pressure test machine (PT-1176, Baoda, China). The adhesion forces in moist environments (relative humidity of 98%) were measured using a humidity tension machine (PT-1176HWHS, Baoda, China). Finally, the adhesion forces in underwater environments were measured using the same computer servo pull pressure test machine, with the sample fully submerged in a petri dish filled with deionized water.

### Fabrication of the skin-attachable electronic device

The photoresist template of the Geckus array was filled with PDMS. Notably, the substrate of the photoresist template is a flat glass with an extremely smooth surface, and its roughness is negligible; thus, it does not introduce additional surface roughness at the contact interface. The thickness of the PDMS layer was controlled at 100 μm via spin coating (first at a speed of 500 rpm for 9 s and then at a speed of 1,500 rpm for 40 s). Then, the cured Geckus array sample was pasted on the surface of a Teflon film, and another Teflon film was pasted on the back of the adhesive sample, which was used as a mask with a rectangular window (area of 1 cm × 2.5 cm). The back of the Geckus array sample was sputtered with SiO_2_ (30 nm) and Cr (20 nm) via the window, and then Au was deposited (100 nm), which was used to generate crack-introduced resistance variations during deformation. The Teflon film mask was then removed, and a conductive tape was attached to the 2 extremities of the Au layer to acquire the sensing signals. Finally, the device was packaged with thin PDMS via spin coating (first at a speed of 500 rpm for 9 s and then at a speed of 2,000 rpm for 40 s). After the packaged PDMS was cured, the device could be easily stripped off the Teflon film.

### Sensing measurements in a low-pressure environment

A remote-controlled manipulator was placed in an airtight acrylic chamber. We used polypropylene tape to fix the goat skin on a finger of the mechanical hand, so that it could deform with the movement of the finger joints of the manipulator. The sensor was attached to the wet goat skin, and the signal lines were taken out of the chamber via 2 holes in the wall of the acrylic chamber (the holes could be closed tightly, and the signal lines were very thin, so that the air tightness of the chamber was not affected). The manipulator was powered by 4 dry batteries (1.5 V) connected in series. A vacuum pump was used to evacuate the chamber until the pressure reached 30 kPa. The inner pressure of the chamber could be stably maintained at 30 kPa due to its good air tightness. The fingers of the manipulator were controlled by external gloves to bend at various angles, and the corresponding resistance changes of the sensor were recorded by the signal generator.

## Data Availability

Data are available on request from the authors.

## Supplementary Materials

Supplementary 1 Figs. S1 to S31 Tables S1 to S4 Movies S1 to S7 References [49–51]
