# Supplementary material for: Bioinspired Reversible Adhesive with High Strength for Wearable Electronics under Diverse Environments
Source: Research (Wash D C). 2026 Jun 4;9:1309. doi: 10.34133/research.1309 (PMC13234439; doi:10.34133/research.1309)
Supplement: Supplementary 1 — Figs. S1 to S31 Tables S1 to S4 Movies S1 to S7 References [49–51] [file research.1309.f1.zip › Revised supplementary materials.docx]

**Supplementary Materials for**

**Bioinspired Reversible Adhesive with High Strength for Wearable Electronics under Diverse Environments**

Yihang Wu^1^, Huiming Liu^1^, Hongmiao Tian^1*^, Xiangming Li^1,2^, Duorui Wang^2^, Bo Sun^1^, Quanyi Zhao^1^, Jinyu Zhang^1^, Tianxiang Lan^1^, Guifang Liu^2^, Xiaoliang Chen^1,2^, Chunhui Wang^1^, Jinyou Shao^1,2*^

1

Micro-and Nano-technology Research Center, State Key Laboratory for Manufacturing Systems

Engineering, Xi’an Jiaotong University, Xi’an, Shaanxi 710049, China.

2

Frontier Institute of Science and Technology (FIST), Xi’an Jiaotong University, Xi’an, Shaanxi 710049, China.

*Corresponding author. Email: hmtian@xjtu.edu.cn

jyshao@xjtu.edu.cn

**Supplementary notes**

**Note 1: Numerical analysis of liquid morphology in microholes during imprinting manufacturing process**

In the imprinting process, the morphology of the liquid in the microholes of template is strongly related to the wetting angle of the liquid. By using the commercial software, Comsol, we explored the process of liquid filling in the microhole of the template under imprinting and compared the morphology difference under polymeric fluid with different wetting angles. The analysis is based on an axisymmetric model of multi-physics including laminar two-phase flow and phase-field. The interface between the polymeric fluid and air is represented by the phase field, which means that the interface is not an absolute liquid-air boundary, but a continuous and variable thin layer. Similarly, the physical parameters in this model, such as viscosity and density, are continuously changed. This interface is implemented through the phase function φ. When the value of the phase field function φ comes to -1, it represents air, and when value comes to 1, it represents liquid, while the gradient change region between -1 and 1 represents the liquid-air interface.

Therefore, the volume fraction of air can be expressed as *V_f1_* = (1 - φ)/2, and the volume fraction of the liquid can be expressed as *V_f2_* = (1 + φ)/2.

Thus, each physical parameter under the phase field model can be obtained as:

$\rho=\rho_{air}V_{f1}+\rho_{liquid}V_{f2}$ (1)

$\eta=\eta_{air}V_{f1}+\eta_{liquid}V_{f2}$ (2)

Where ρ presents the mass density of fluid, and η presents the hydrodynamic viscosity, among the equations, subscript “air” presents the relevant parameters of air; subscript “liquid” presents the relevant parameters of the liquid.

According to the mass conservation property, the convection-diffusion equation of the phase function φ can be expressed in the form of the Cahn-Hilliard equation^[49]^:

$\frac{\partial\phi}{\partial t}+\nabla\cdot(u\phi)=\nabla\cdot(\mu\nabla G)$ (3)

Where *u* presents the velocity of the fluid (including liquid and air), μ presents the fluid mobility, and G presents the chemical potential.

The rheological state of fluid can be described by Navier-Stokes equation^[50,51]^:

$\left\{\begin{matrix} \rho\frac{\partial u}{\partial t}+\rho u\cdot\nabla u=-\nabla p+\eta\nabla^{2}u+\frac{1}{3}\eta\nabla(\nabla\cdot u)+F_{st}+\rho g \\ \frac{\partial(\rho u)}{\partial t}+\nabla\cdot(\rho u)=0 \end{matrix}\right.$ (4)

Here, *u* represents the fluid velocity, *p* represents the internal pressure of the liquid, and g represents the gravity acceleration.Among them, the *F*_st_ and *ρg* represent the effects of surface tension, gravity, respectively.

In this model, the height of the template microhole is 30 μm and the width is 30 μm. Other relevant simulation parameters are shown in Supplementary Table 1.

**Note 2: Numerical analysis toward the contact-splitting behaviors of Geckus on flat surface**

1. Geometric modeling:

To understand the adhesion mechanism of adhesives in dry and under water environment, the commercial simulation software ABAQUS was employed to analyze the adhesion behavior. Here, a cohesive zone model based on the cohesive surface was adopted to simulate the contact and separation processes of the mushroom-shaped microstructures. During the pressing/attachment process, the general contact was used to simulate contact. During the pulling/detachment process, the cohesive contact property was introduced. The formula of the cohesive constitutive law can be depicted as follows:

$F=\left\{\begin{matrix} F_{n} \\ F_{s} \\ F_{t} \end{matrix}\right\}=A\left[\begin{matrix} K_{nn} & K_{ns} & K_{nt} \\ K_{ns} & K_{ss} & K_{st} \\ K_{nt} & K_{st} & K_{tt} \end{matrix}\right]\left\{\begin{matrix} \delta_{n} \\ \delta_{s} \\ \delta_{t} \end{matrix}\right\}=AK\delta$ (5)

where *F_n_* denotes the normal separation force during the detachment; *F_s_* and *F_t_* represent two shear separation forces, respectively; *A* is the total area of the interface; *K* stands for the interface stiffness matrix; *δ_n_*, *δ_s_*, *δ_t_* are opening displacements of the cohesive interface in three directions. The maximum nominal stress damage criterion was adopted to control the damage of the interface, and its constitutive formula can be presented as:

$Max\left\{\frac{t_{n}}{t_{n\max}},\frac{t_{s}}{t_{s\max}},\frac{t_{t}}{t_{t\max}}\right\}=1$ (6)

The geometric model was a two-dimensional model, which mainly includes the Geckus microstructure and Si wafer (see Fig. S1). The Fig. S1 illustrates the dimensions and layout of a Geckus structure. The original dimensions are as follows: length (*l_1_*) is 90 microns, height (*h*) is 40 microns, the thickness of the Geckus structure's top (*t_0_*) is 25 microns, the thickness of its bottom (*t*) is 5 microns, the length of the side (*L_s_*) is 10 microns, the length of the top (*L_t_*) is 35 microns, and the width of the bottom (*W*) is 30 microns. These dimensions provide the specific geometric details of the structure. Parameter optimization simulation of the structure is carried out by controlling the brim thickness (*t*), brim length (*L_t_*) of the Geckus structure.

2. Boundary conditions and Materials:

During the attachment and detachment process, the Si wafer is fully immobilized. A reference point (RP) is arranged at the center of the top surface of the mushroom-shaped microstructure, and the top surface of the Geckus microstructure is coupled with the reference point. By applying the displacement along the Y direction at the reference point, the Geckus microstructure can be contacted and separated from the Si wafer.

In ABAQUS, set the Si wafer as a rigid body. For the Geckus microstructure base made of PDMS, use the Neo-Hookean model with C10 = 3.3e-7 and D1 = 30000. No material properties are needed for the Si wafer; just define it as a rigid body in the geometric model.

The top surface of the Si wafer was selected as the main surface and the bottom surface of the Geckus microstructure was the secondary surface because the stiffness of the Geckus microstructure is relatively smaller than that of the Si wafer. The maximum nominal stress damage criterion is used as the initial damage criterion of the interface, and the corresponding parameters are both 0.27 MPa. The interface stiffness is 1 N mm^-1^. The triangle diagram for the cohesive mode is demonstrated in Fig. S2.

3. Apply negative pressure:

The geometric model is designed with symmetry to enhance computational efficiency. By applying pressure at key locations (Press-Inner, Press-Out, and Press-Bot) within the microstructure cavity, its deformation and behavior can be effectively simulated and controlled. The initial pressure is set to the standard atmospheric pressure of 1.013×10⁵ Pa. During the simulation, the Y-direction displacements of three critical points at the cavity's bottom (H0, StartPoint, and EndPoint) are monitored in real-time to track the microstructure's deformation. These displacement data are then used to calculate the volume change ratio of the cavity before and after deformation. Moreover, the applied pressure is dynamically adjusted using the UAMP user-defined load feature in the ABAQUS software. This approach enables precise simulation of the negative pressure experienced during the cavity's compression and peeling processes.

During the program execution: At the Initial position, the Press-Inner, Press-Outer, and Press-Bot surfaces are all at atmospheric pressure. At the Preload position, the Press-Outer surface remains at atmospheric pressure. The Press-Bot surface, in contact with the ground, is at 0 pressure. The Press-Inner surface pressure depends on the cavity volume, calculated via formulas 3 and 4 to get *V_Inner_*. The Pull-1 stage follows the same calculation process as the Preload stage. At the Pull-2 stage, the Press-Outer surface is at atmospheric pressure. The Press-Inner surface pressure matches the Preload stage. The Press-Bot surface pressure equals the Press-Inner pressure due to the StartPoint node detaching. At the End stage, all surfaces return to atmospheric pressure, mirroring the Initial stage. See Supplementary Table 2 for detailed calculations.

$V_{Initial}=W\times(t_{0}+t)-\frac{1}{2}\pi R^{2}$ (7)

$V_{current}=W\times(t_{0}+t-H_{0})-\frac{1}{2}\pi R^{2}$ (8)

$V_{Inner}=\frac{V_{current}}{V_{Initial}}\times1.013\times10^{5}$ (9)

4. capillary force:

In this study, the capillary force is calculated using the following formula:

$F_{\text{pressure}}=-\pi R^{2}\gamma_{LV}\frac{\cos\theta_{1}+\cos\theta_{2}}{h}$ (10)

where: *R* is the radius of the microstructure cavity, *h* is the height of the liquid column, *γ_LV_* is the surface tension coefficient between the liquid and vapor, *θ_1_* and *θ_2_*are the contact angles.

This formula comprehensively considers the geometric parameters of the microstructure cavity, the surface tension of the liquid, and the contact angles to calculate the capillary force under different conditions.

In the numerical simulation, the capillary force is applied at the bottom of the structure using the UAMP user-defined subroutine in ABAQUS. The calculated capillary force based on the above formula is input as a load in the model to simulate the effect of capillary force on the microstructure. The Fig. S5 illustrates the distribution of capillary force on the microstructure cavity. In this diagram, the arrows indicate the direction and distribution of the capillary force applied at the bottom of the microstructure cavity. This visualization helps in understanding how the capillary force is distributed across the structure and aids in analyzing its impact on the overall behavior of the Geckus structure.

5. Structural optimization:

In this study, parameter-optimized simulations of the Geckus structure were carried out by controlling the edge thickness (*t*) and edge length ((*L_t_*) of the structure. The aim of the optimization is to improve the overall stability and performance of the Geckus structure when capillary forces or other external loads are applied. Supplementary Table 3 shows the structurally optimized dimensional parameters.

**Note 3: In-situ observation system and the observation process**

The interfacial states of different structures in the contact splitting process were observed by our self-built in-situ observation system. Specifically, the system is mainly composed of an optical microscope, a force sensor, a displacement platform, a leveling platform, the sample placement, the bracket and the glass (Fig. S20). The specific observation process are as follows:

1. Fix the structure patch on the sample placement and adjust the leveling platform until the patch surface is parallel to the glass surface;

2. Lift the displacement platform until the force sensing value reaches the initial preload value;

3. Drop the displacement platform, at the same time control the focuses of the optical microscope and take relevant photos.

Especially, in the interfacial observations of wet environments, we need to drop liquid on top of the sample in advance. Due to the cracks appear as white color, which is nearly the base color of the entire interface when using pure water as the liquid environment. Hence we drop blue ink in the deionized water as the liquid environment so that the observations of the specific interfacial separation would be more clearly. It is worth noting that the contact angles of deionized water and deionized water with added blue ink on both the glass and the Geckus patch surfaces are nearly identical (Fig. S7); therefore, the addition does not alter the properties or wetting behavior of deionized water and does not affect the observed detachment process.

**Supplementary images**

**
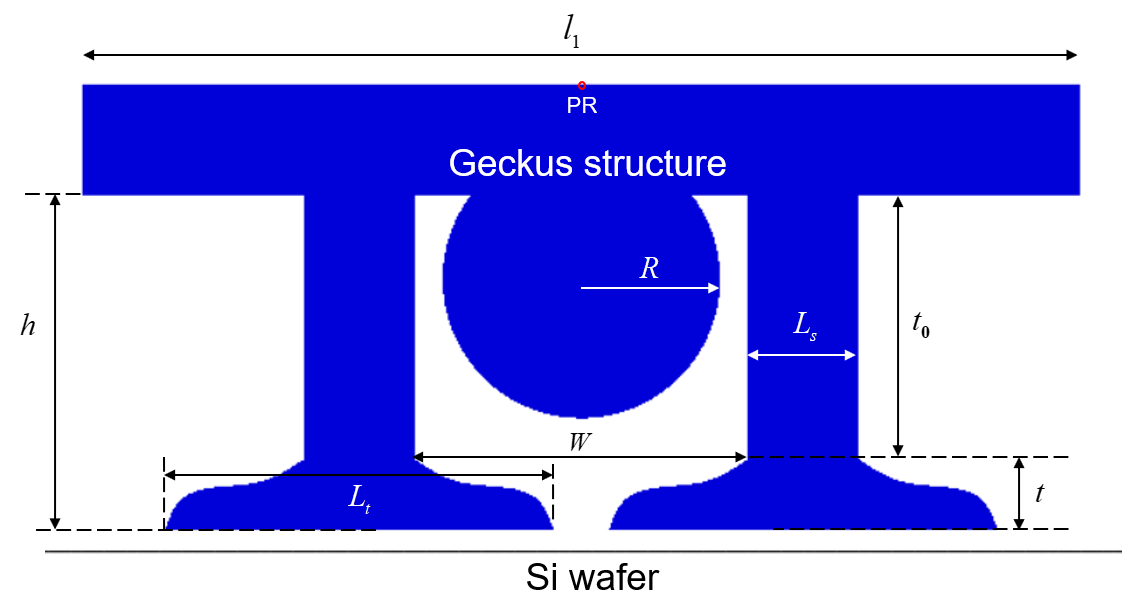
**

**Fig. S1.** Schematic diagram of geometric model of the numerical simulation. The geometric model was a two-dimensional model, which is mainly composed of the Geckus microstructure and Si wafer.

**
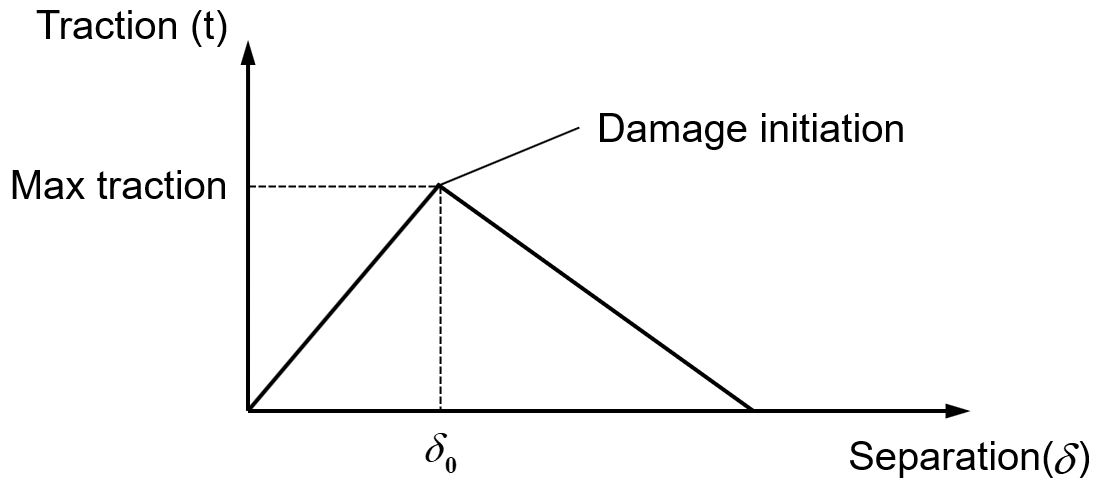
**

**Fig. S2.** The triangle diagram for the cohesive mode.


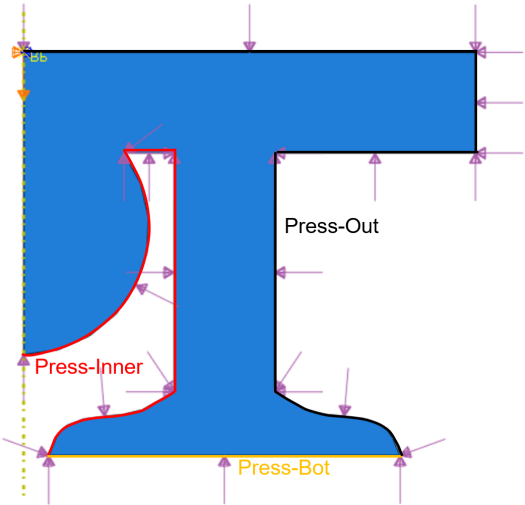

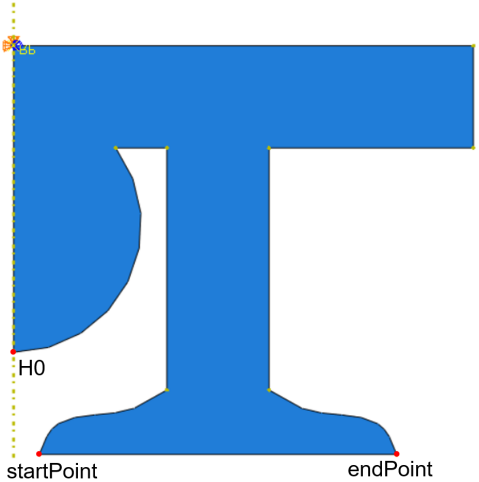


**Fig. S3.** Schematic diagram for applying negative pressure. Press-Inner, Press-Out and Press-Bot represent the pressure inside the Geckus microstructure chamber, the pressure outside the chamber and the pressure at the bottom of the chamber, respectively. The Y-direction displacements of the three point sets H0, startPoint and endPoint at each time step are output in real time.


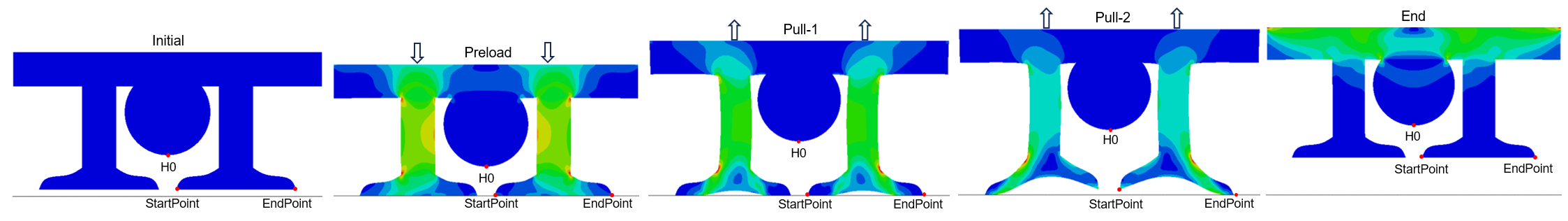


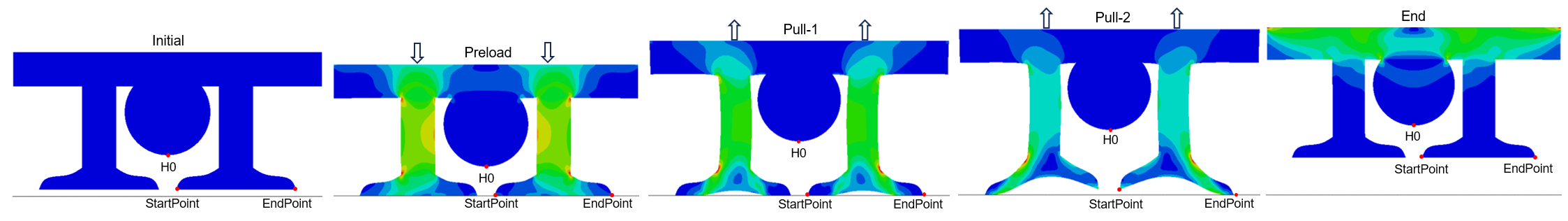


**Fig. S4.** Schematic calculation of pressure at five characteristic positions. Press-Inner, Press-Outer and Press-Bot pressures are calculated by obtaining the displacements of H0, StartPoint and EndPoint at five feature positions: Initial, Preload, Pull-1, Pull-2 and End.

**
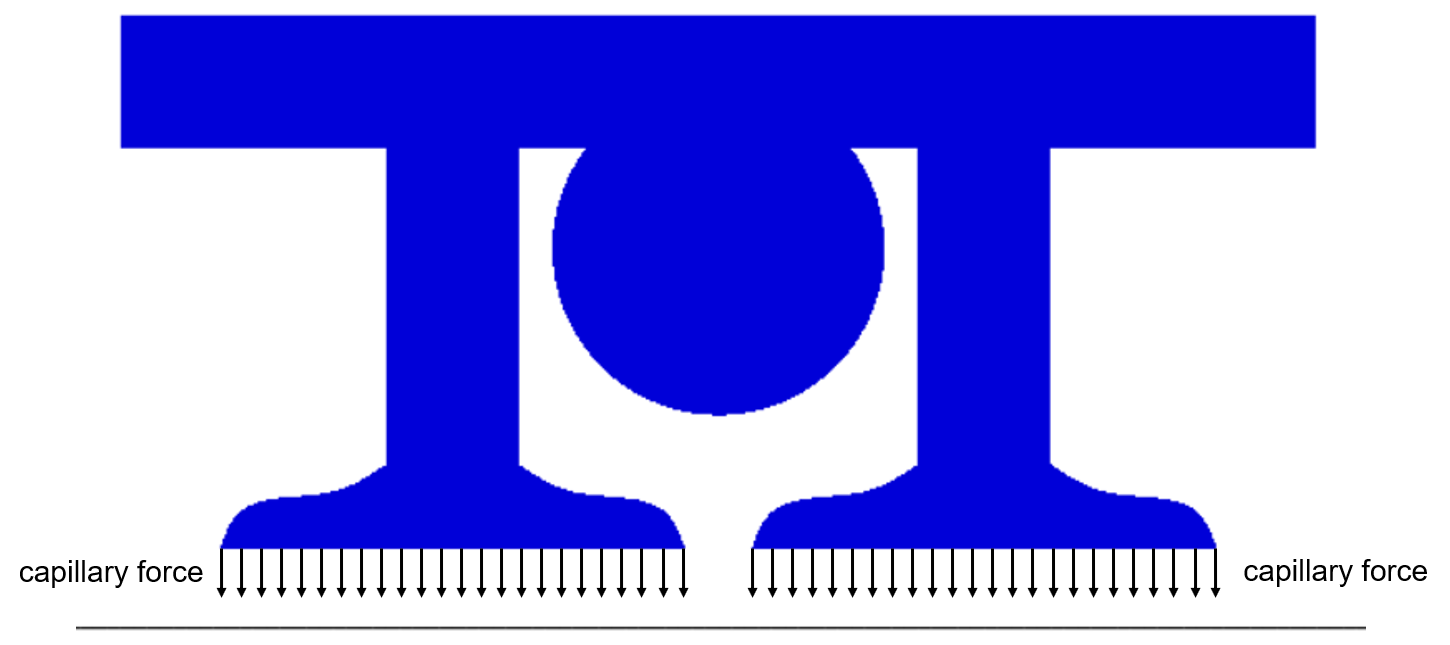
**

**Fig. S5.** Schematic diagram of capillary force distribution on a microstructure cavity. The arrows indicate the direction and distribution of the capillary force applied at the bottom of the microstructure cavity.


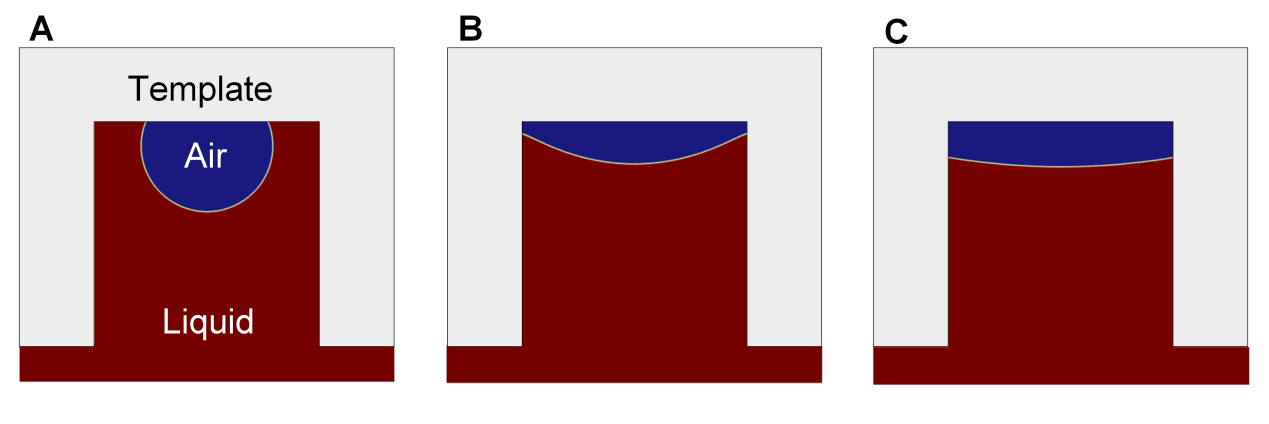


**Fig. S6.** Different morphology of trapped-liquid with different wetting angles. (A) The wetting angle of the liquid on the template is 65°, (B) 70° and (C) 80°. The shapes of the trapped-liquid in the microholes of the template are closely related to its wetting angles on the template material. In this simulation model, we tested three different contact angles, respectively 65°, 70° and 80°. We found that as the contact angle decreased, the shape of the microdome become more pronounced. Especially when the contact angle come to 65°, the liquid can wetting along the template wall until to the top of the template, forming a more spherical microdome.


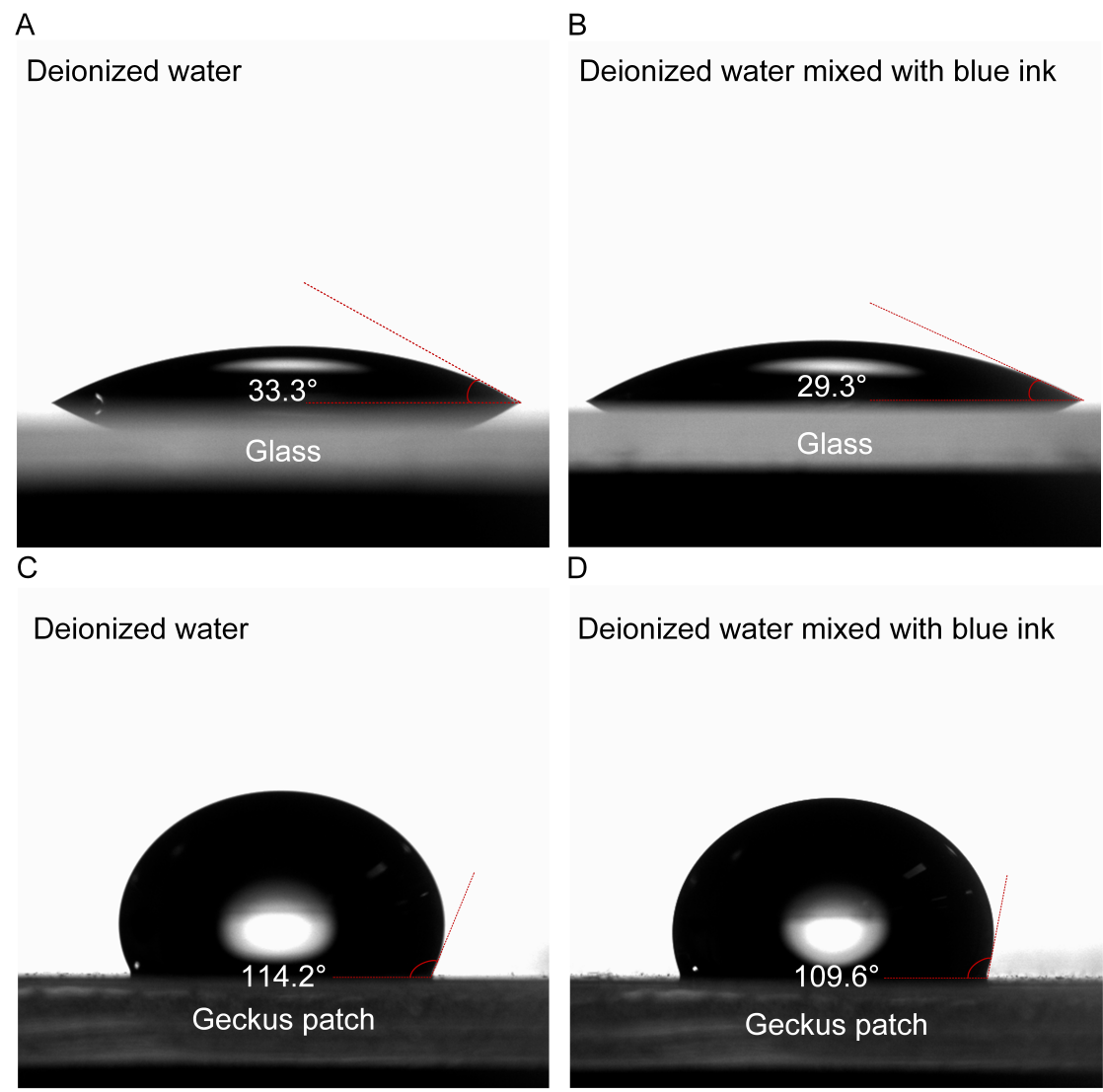


**Fig. S7.** Characterization of wetting angles. Contact angles of (A) deionized water and (B) deionized water with added blue ink on glass; contact angles of (C) deionized water and (D) deionized water with added blue ink on the Geckus patch. The differences in contact angles between the two liquids on the glass and the Geckus patch surfaces are very small, indicating that the addition of blue ink does not alter the properties or wetting behavior of deionized water.


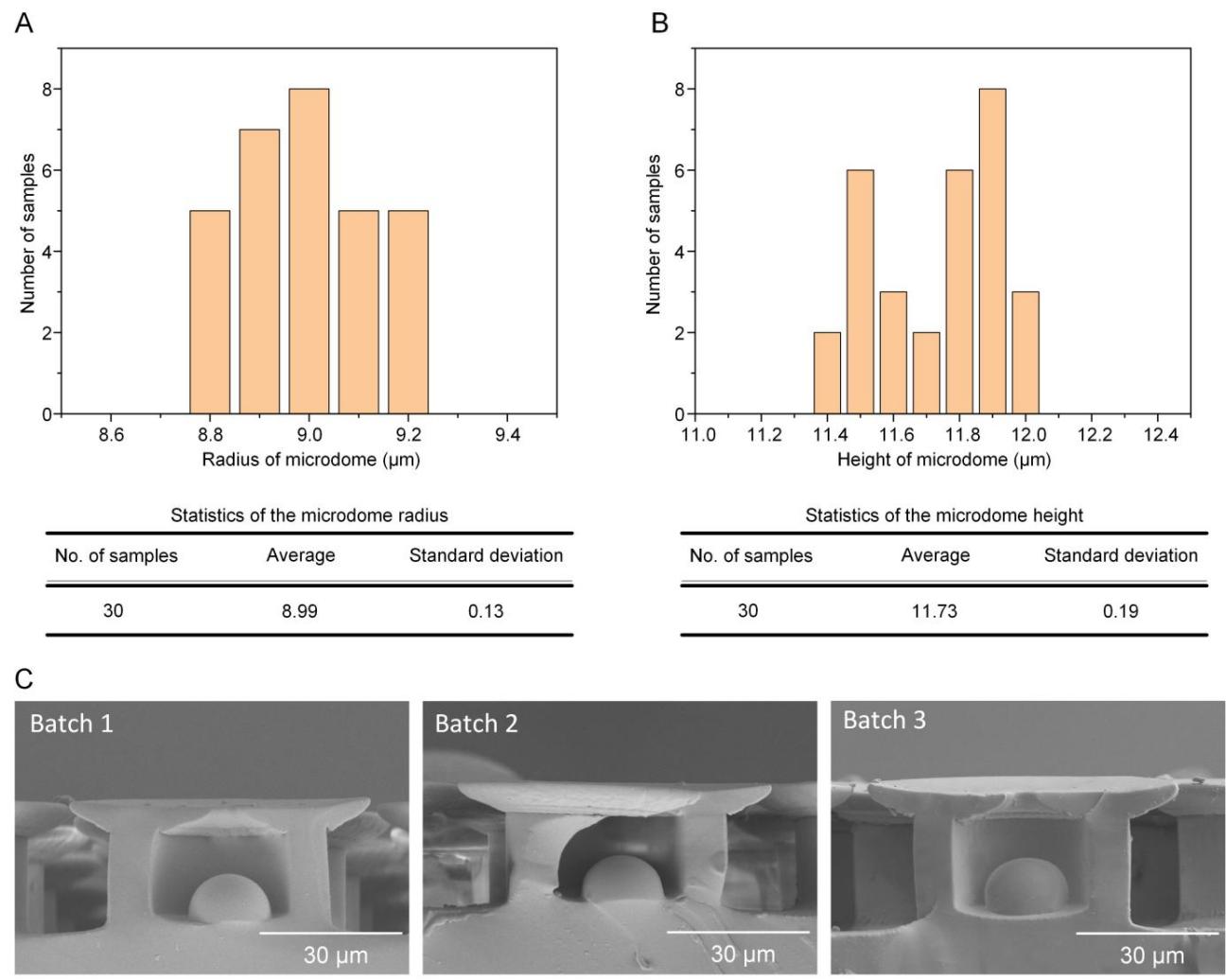


**Fig. S8.** Characterization of the morphological uniformity of microdomes. (A) Radius statistics and (B) height statistics; (C) SEM cross-sectional images of three batches. The results indicate that the radius and height of the micro-domes are fairly uniform, with good batch-to-batch consistency in structural morphology.


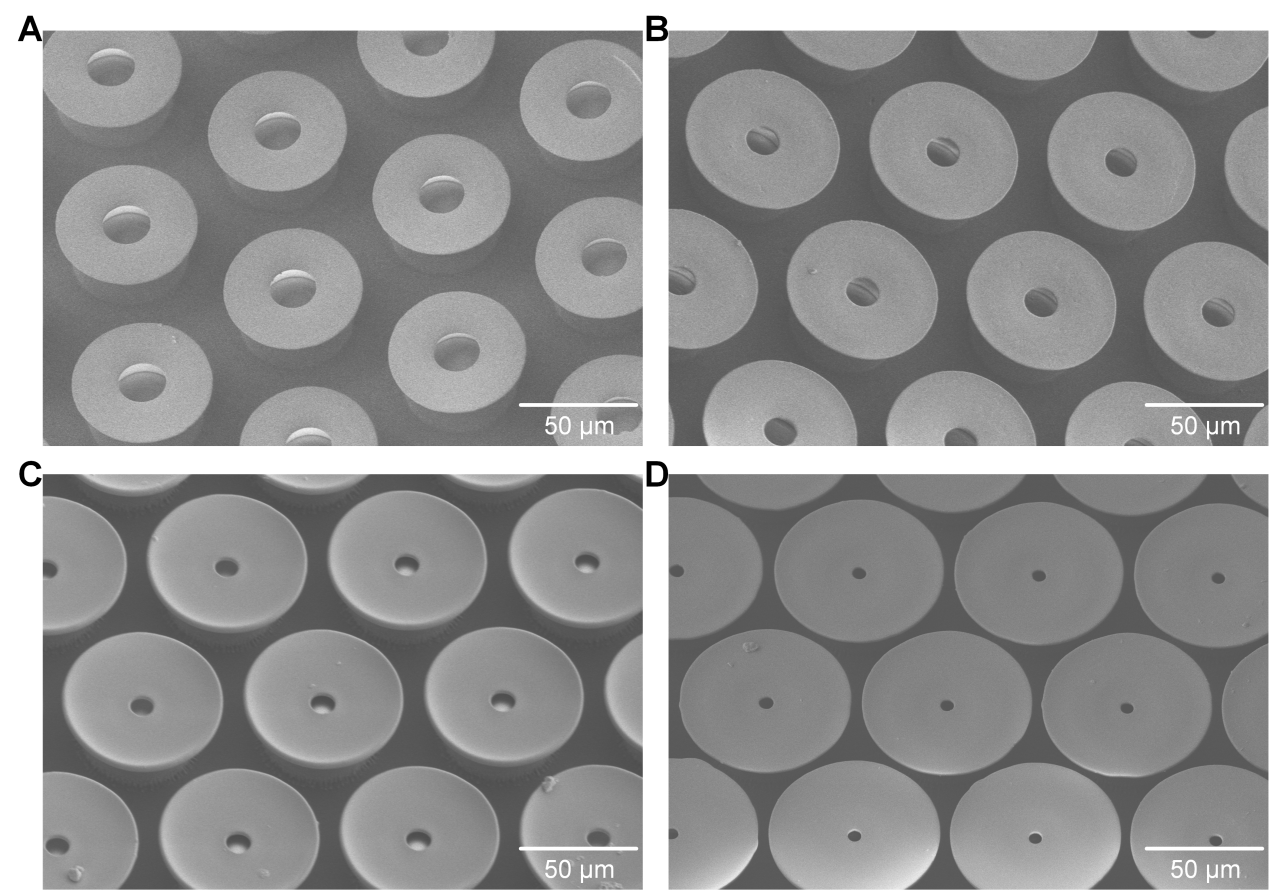


**Fig. S9.** SEM images of Geckus structures with different tip sizes.The sizes are (A) 20-60 μm, (B) 15-65 μm, (C) 10-70 μm, and (D) 5-75 μm. When describing the sizes of the tips, we use the first number to represent the inner diameter of the tips, and the second number to represent the outer diameter of the tips.


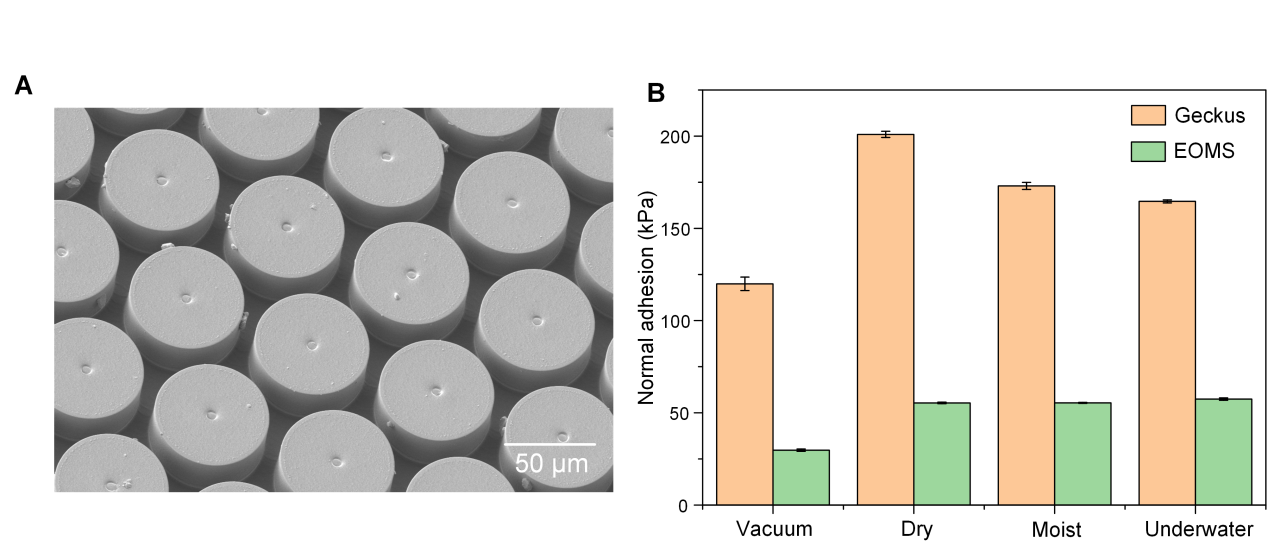


**Fig. S10.** Characterization of EOMS sample (The terminal size is 5–75 μm). (A) The SEM image of EOMS. (B) Comparison of the normal adhesion strength of Geckus patches and EOMS under various environments (with a preload of 50 kPa). The normal adhesion of the Geckus are significantly higher than the EOMS.


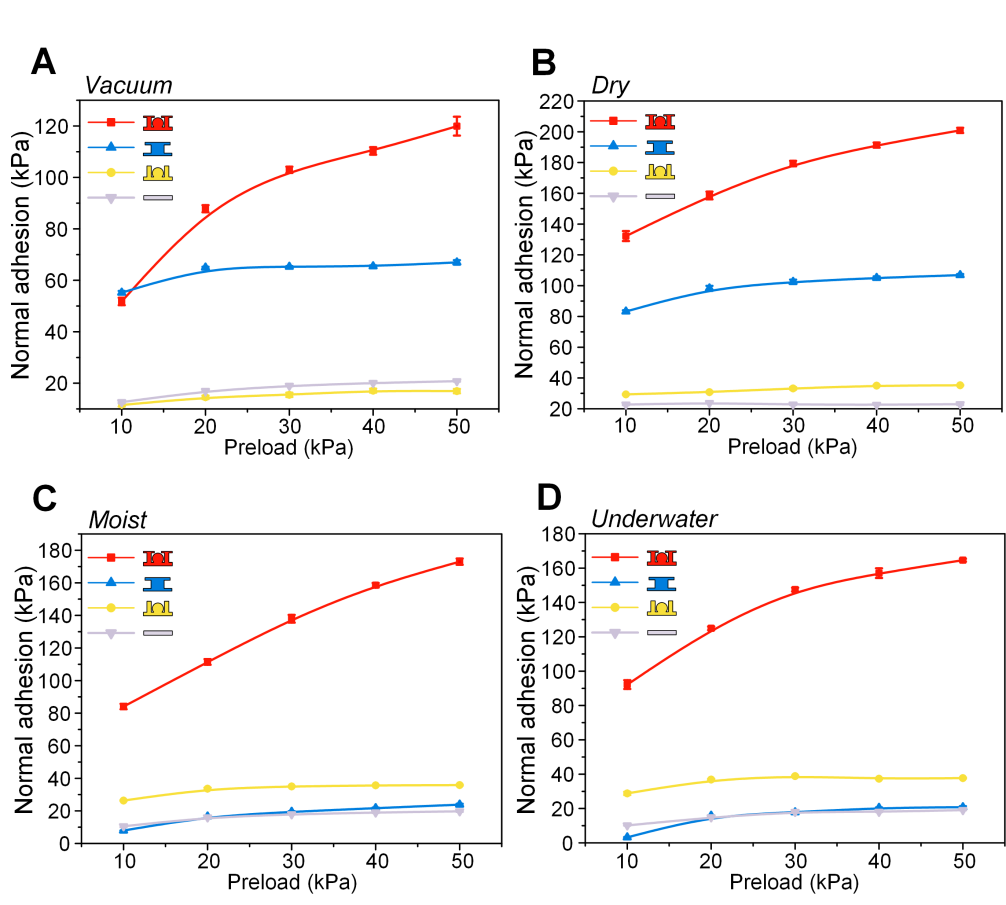


**Fig. S11.** Characterization of the adhesion forces of the Geckus structure, MSAM, EOMS, and the flat under various environments. (A-D) Normal adhesion strengths of the different structures as a function of the preload in vacuum, dry, moist, and underwater environments, respectively. The normal adhesion is all increased with the preload.

**
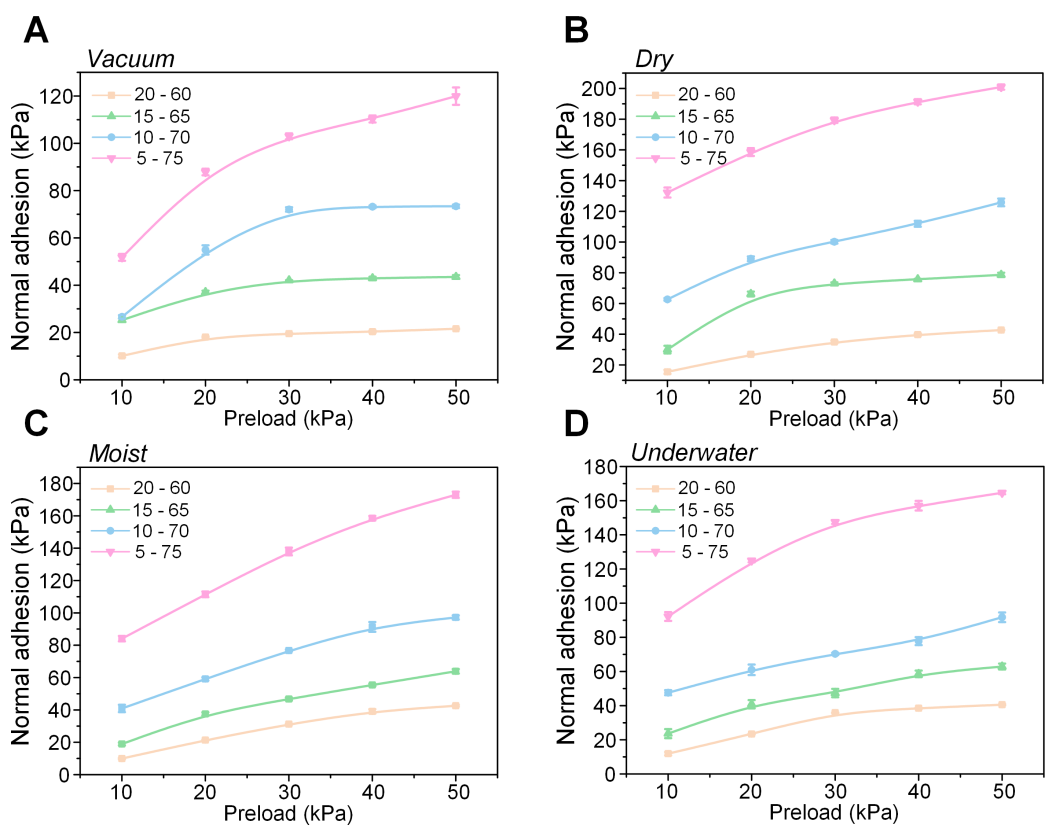
**

**Fig. S12.** Characterization of the adhesion forces of the Geckus structures with different tip sizes under various environments. (A-D) Detailed curves toward normal adhesion strengths of the Geckus with different tip sizes (20-60 μm, 15-65 μm, 10-70 μm, and 5-75 μm) as a function of the preload in vacuum, dry, moist, and underwater environments, respectively. The normal adhesion of the Geckus samples with different head sizes increases with the preload.


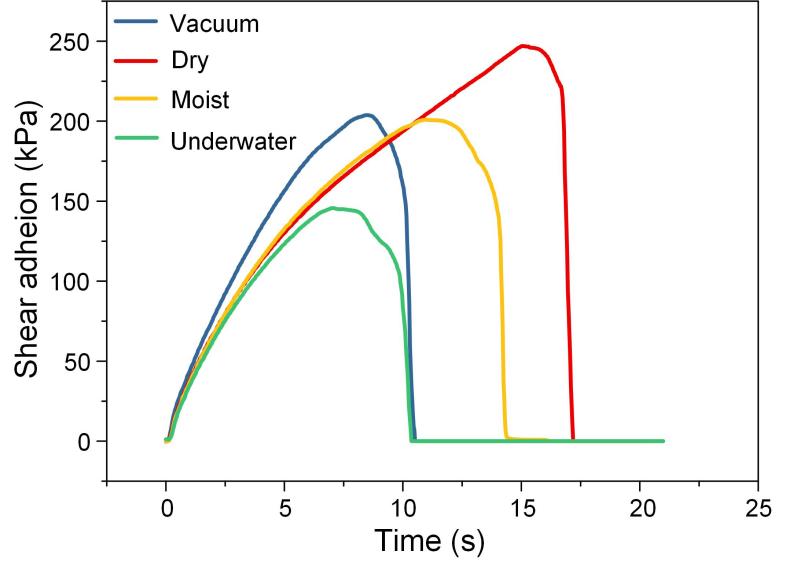


**Fig. S13.** Shear adhesion of the optimized Geckus patch on a silicon wafer. The Geckus patch achieved shear adhesion strengths of 204 kPa (vacuum), 247 kPa (dry), 201 kPa (moist), and 145 kPa (underwater). In vacuum, dry, and moist conditions, the shear adhesion exceeded the normal adhesion values.


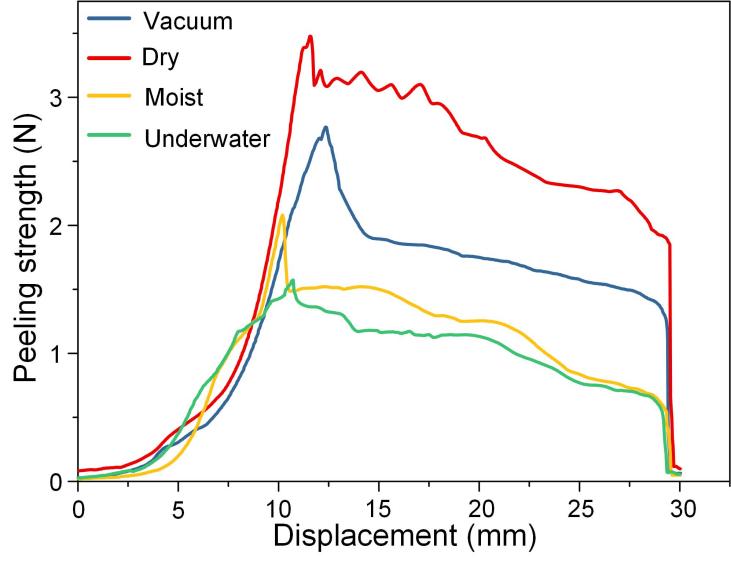


**Fig. S14.** Peeling strength of the Geckus patch on a silicon wafer in four environments. The 3 × 3 cm² patch showed a peak force at initial detachment, followed by decay. Peeling forces in moist and underwater conditions were lower than in vacuum and dry conditions due to water wetting.


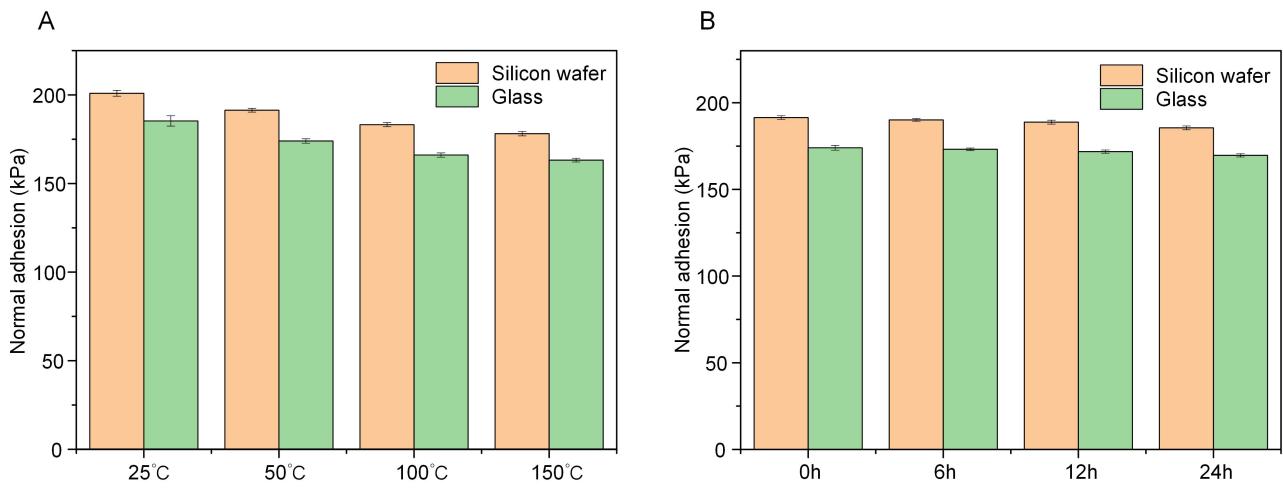


**Fig. S15.** Characterization of the temperature stability of the Geckus adhesive. (A) Temperature dependence of the normal adhesion of the Geckus patch on silicon wafer and glass surfaces (25–150 °C). (B) Adhesion forces measured on wafer and glass surfaces at 50 °C after 0, 6, 12, and 24 h. The adhesive still maintains a strong adhesion force at 150 °C. After heating at 50 °C for 24 h, the adhesion force shows almost no change, demonstrating excellent temperature stability.

**
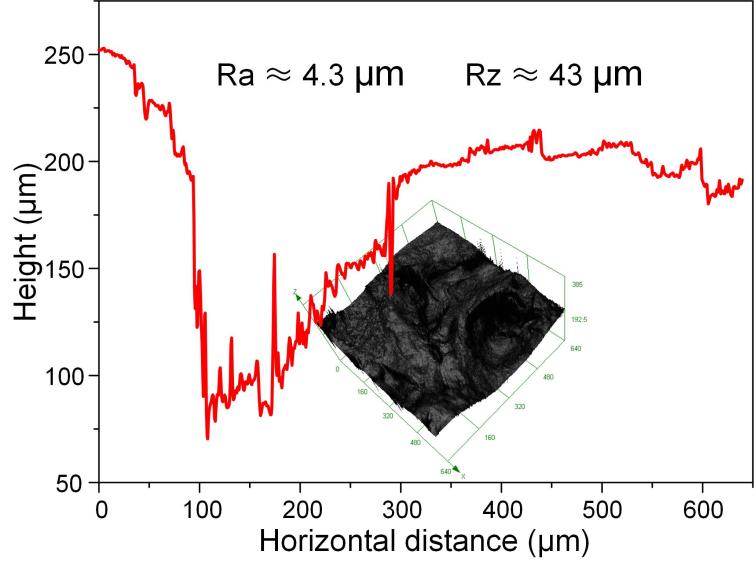
**

**Fig. S16.** Surface roughness of the goat skin that characterized by confocal scanning. Goat skin was used as a human skin replica, with a mean roughness (Ra) of approximately 4.3 μm and a maximum height (Rz) of approximately 43 μm


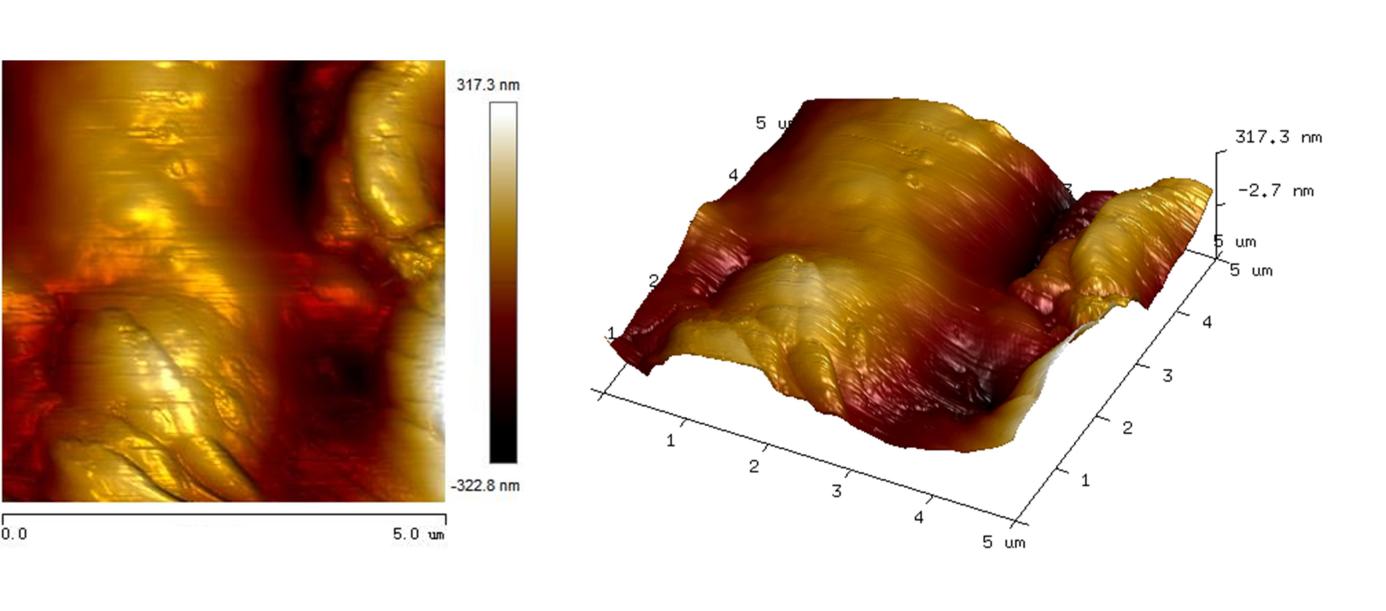


**Fig. S17.** Atomic force microscopy (AFM) topography of the goat skin surface. The image was acquired over a 5 μm × 5 μm scan area. The surface height variation ranges from −322.8 nm to +317.3 nm, with a root mean square roughness (Rq) of 92.7 nm.

**
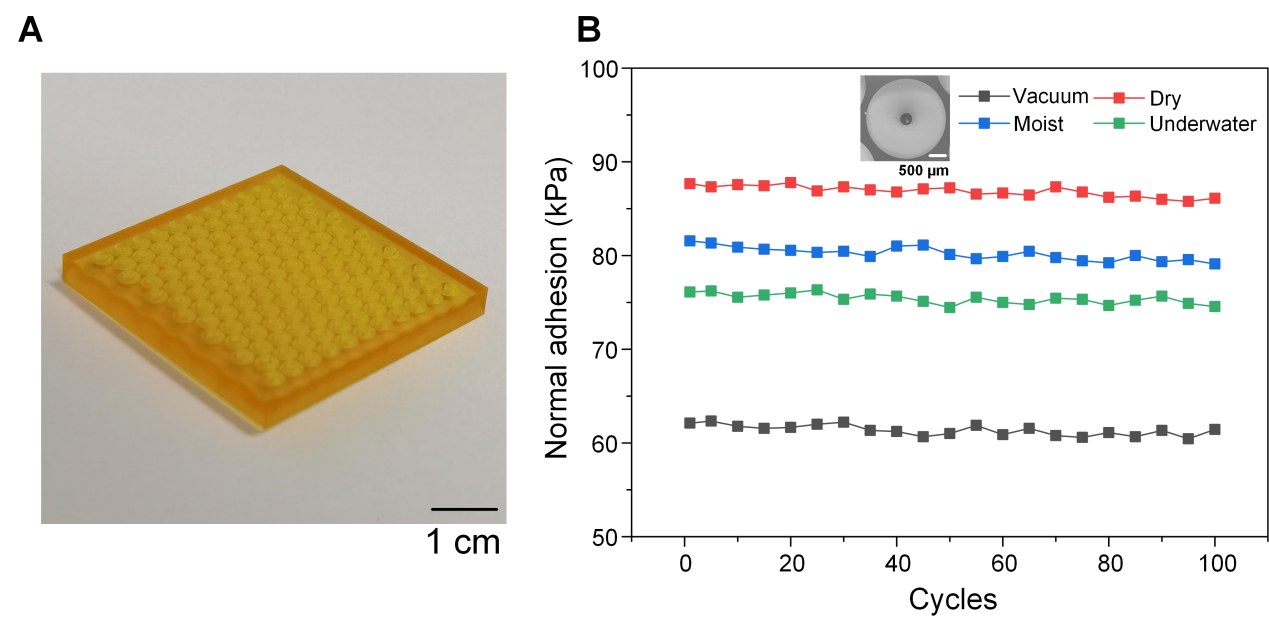
**

**Fig. S18.** Fabrication and characterization of the scaled-up Geckus (Magnified by a factor of 40). (A) The reversed mold of the scaled-up Geckus; (B) The normal adhesion data of the enlarged Geckus under repeat adhesion tests (100 cycles) in vacuum, dry, moist and underwater conditions. The scaled-up Geckus patch demonstrated good repeatability in normal adhesion under vacuum, dry, moist and underwater conditions, but exhibited a decline compared to original-scale structure due to size effect.


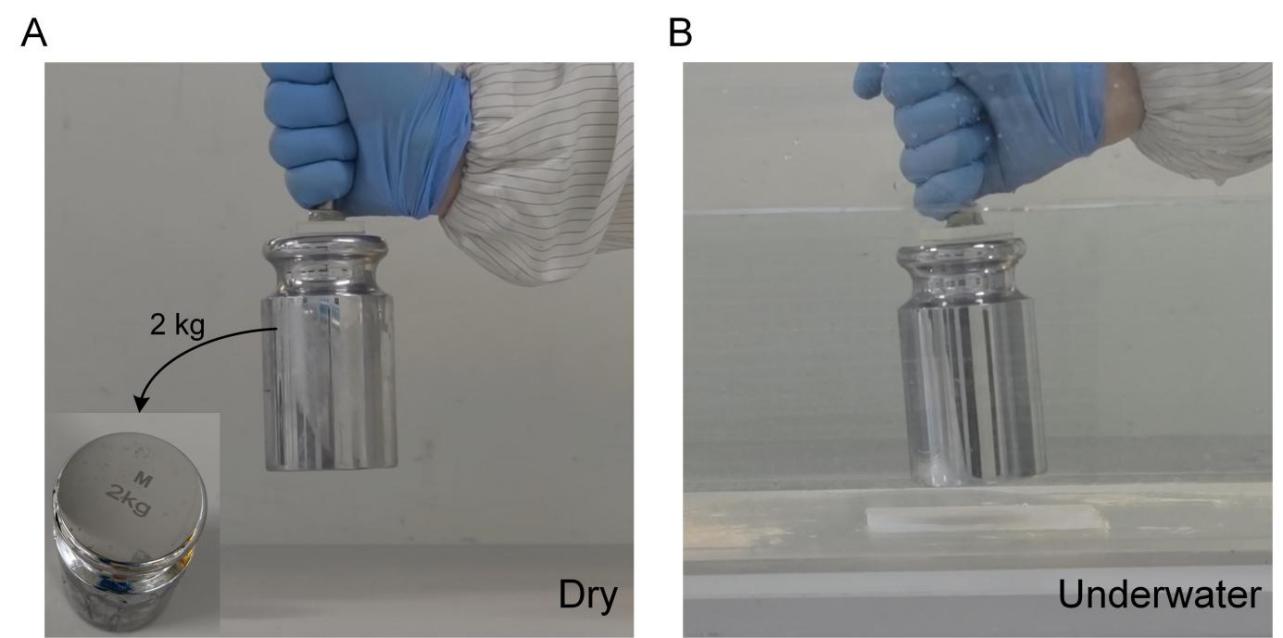


**Fig. S19.** Geckus patch (area 3 cm × 3 cm) grasping a 2 kg weight. (A) Dry conditions. (B) Underwater conditions. The Geckus patch exhibits high load-bearing performance for lifting a mass.

**
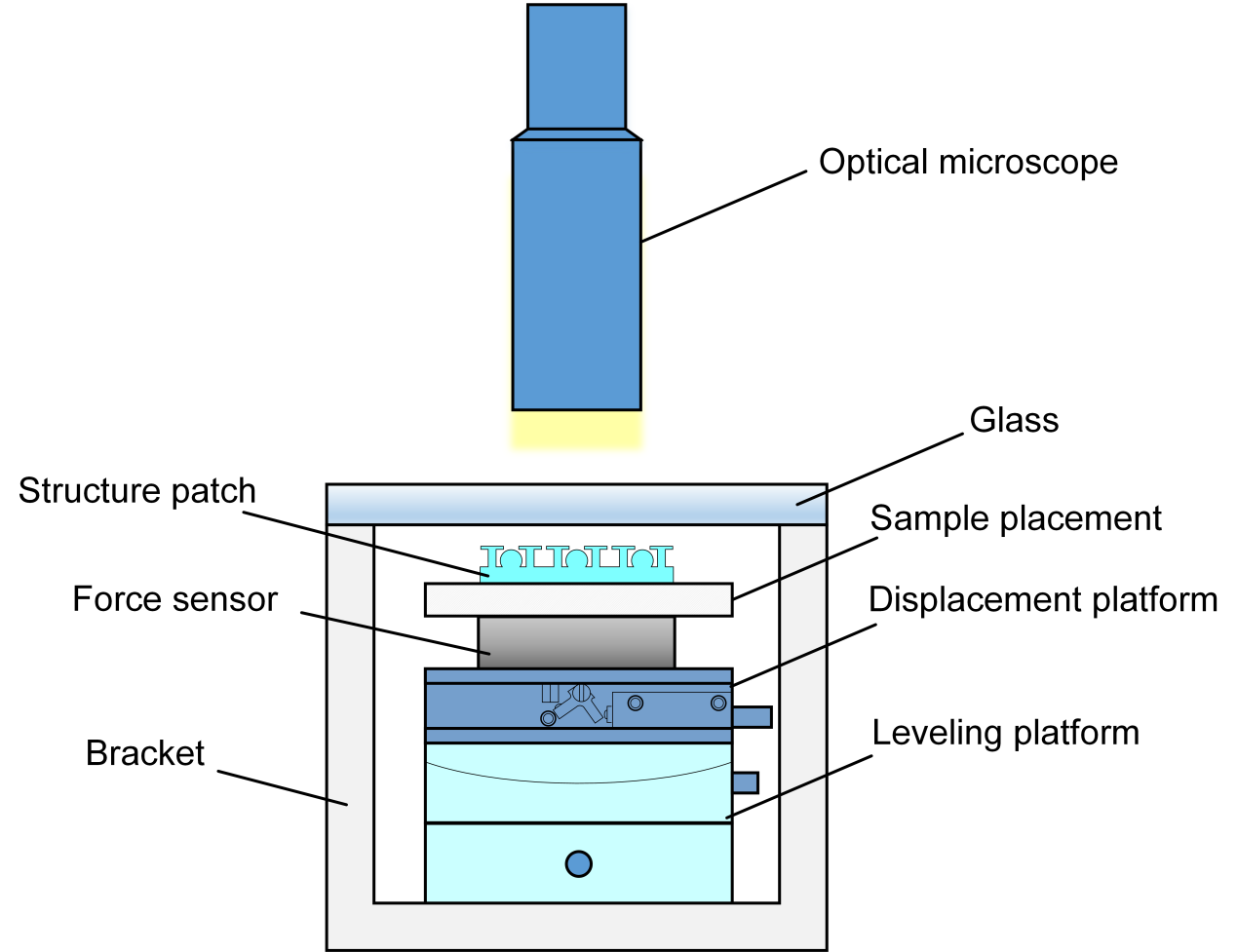
**

**Fig. S20.** Schematic diagram of the in situ observation system. The system is compound with the optical microscope, the sample placement, a force sensor, the displacement platform and the leveling platform.The sample is fixed on the sample placement. After adjusting the leveling platform to make the sample surface parallel to the glass, the displacement platform is regulated to apply preload and initiate detachment, while the interface behaviors are observed synchronously through an optical microscope.

**
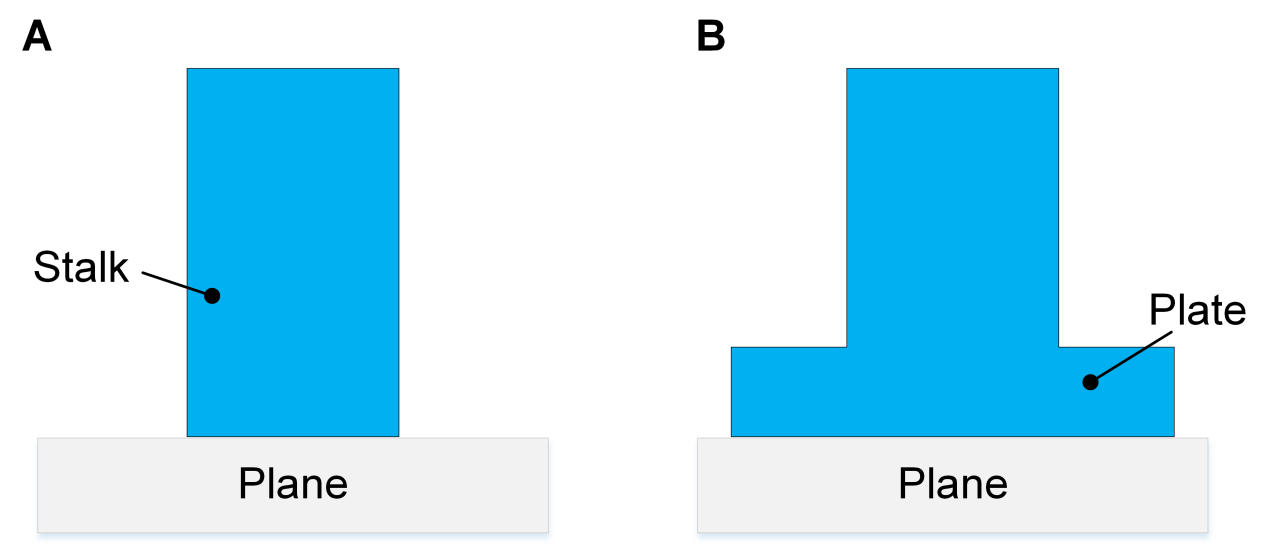
**

**Fig. S21.** Interface contact modes^[39]^. (A) The stalk–plane contact mode. (B) The plate–plane contact mode. The stalk–plane contact mode would form the stress singularities at the edge of the contact interface, i.e., stress concentration phenomena, which manifests as the cracks are highly likely to form at this location and rapidly propagate to the interior. Therefore, the interface adhesion under this contact mode is prone to failure, with low interface strengths and lead to low adhesion force. Different from above, when come to the plate–plane contact mode, the stress in the entire contact area is flattened, which means the stress singularity at the new edge is effectively weakened. Since the stress at the edge is less than the internal, cracks are more likely to form in the central of the interface. The contact stress at the stress singularity point is infinite, while the contact stress at the crack initiation point within the interface is limited, so it can be assumed that the “plate–plane” contact mode is less prone to forming the cracks and has higher interface strengths and greater adhesion forces compared to the “stalk–plane” contact mode.


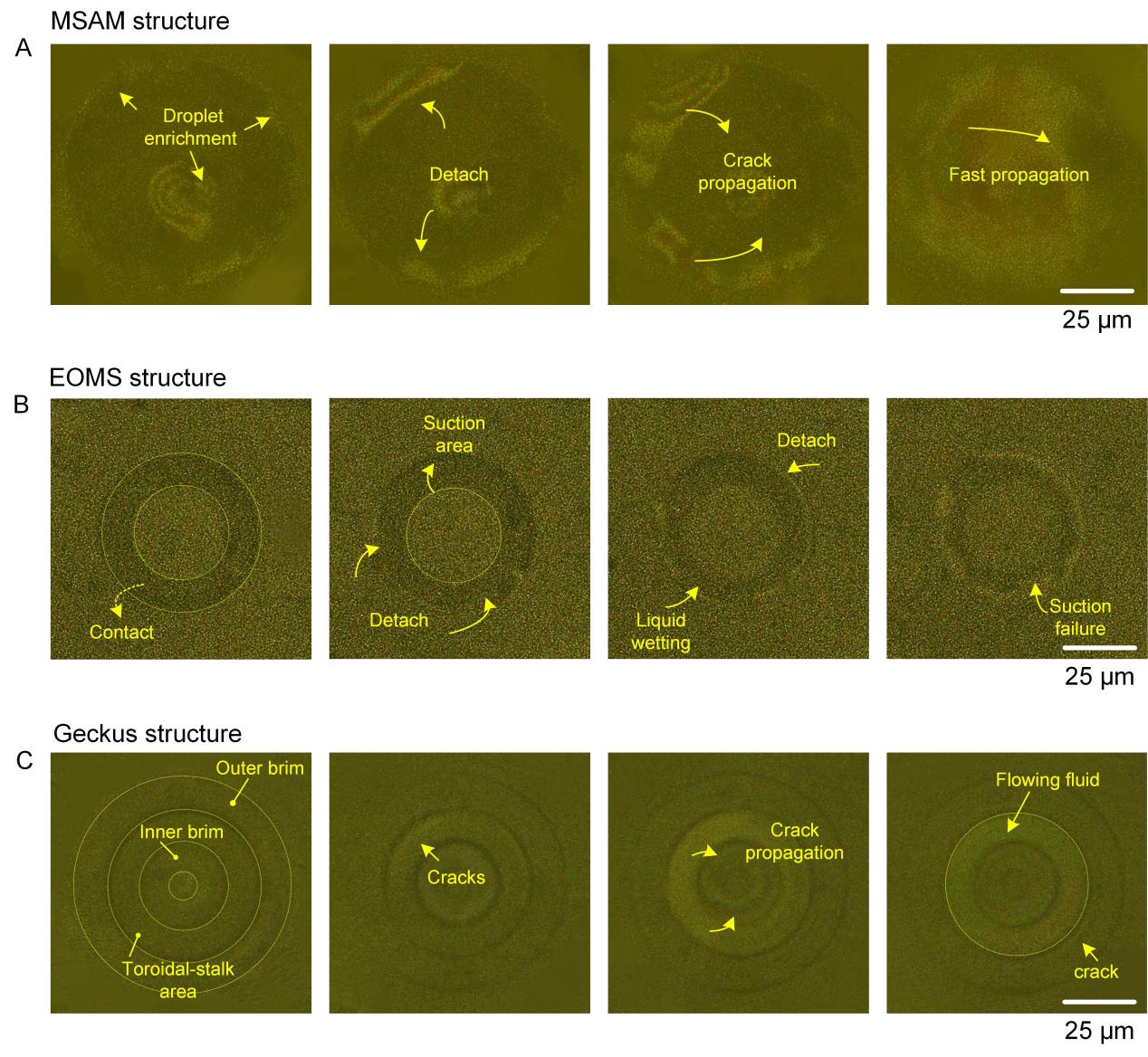


**Fig. S22.** Images of crack nucleation and propagation during underwater detachment for three structures: (A) MSAM, (B) EOMS, and (C) Geckus. These images show crack nucleation and propagation behaviors consistent with those observed in Fig. 4, confirming the reproducibility and reliability of the observations.

**
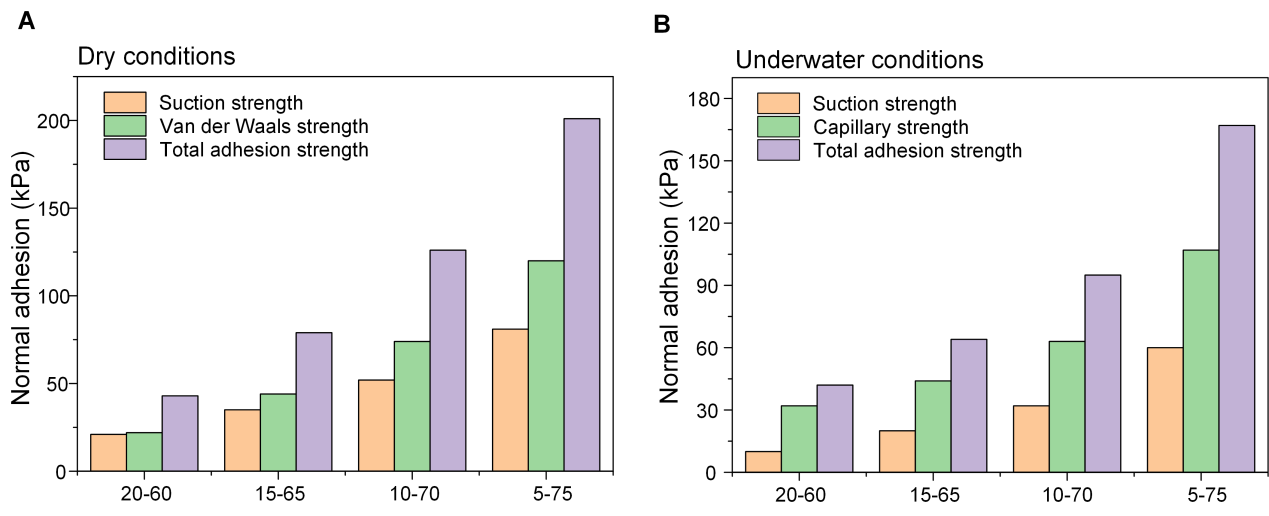
**

**Fig. S23.** Contributions of different forces in the total normal adhesion. (A) The contributions of suction and van der waal forces in the adhesion of dry environments; (B) The contributions of suction and capillary forces in the adhesion of underwater environments. Under dry conditions, van der Waals forces contribute more significantly to adhesion, whereas in underwater environments, capillary forces relatively dominate. Both interactions exhibit an increasing magnitude with the enlargement of the tip size.


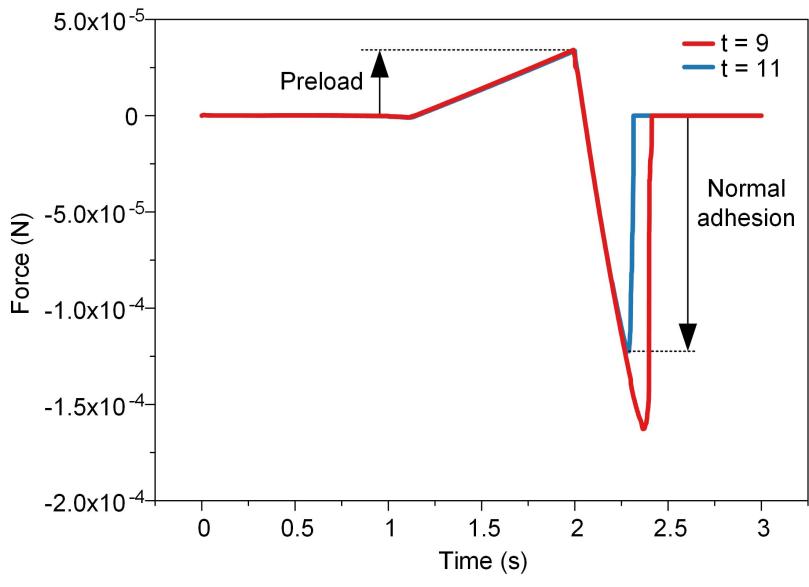


**Fig. S24.** Time-dependent adhesion profiles for the normal adhesion of Geckus models (tip thickness t = 9 μm and 11 μm). Under the same preload, the adhesion force of the structure with the crack reentry behavior (t = 9 μm) is higher than that of the structure without this behavior (t = 11 μm), indicating that the crack reentry phenomenon can enhance adhesion performance.


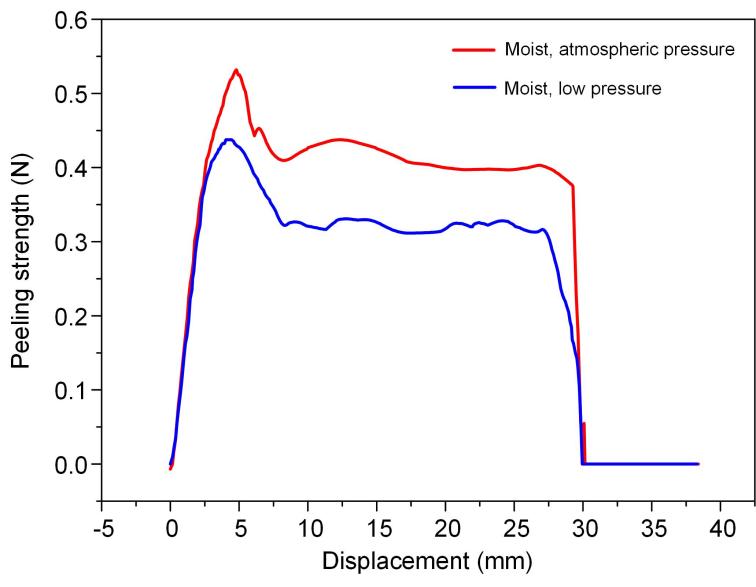


**Fig. S25.** Peeling force of the Geckus adhesive on moist goat skin under ambient pressure and low pressure (30 kPa). The tests were conducted to evaluate the interfacial adhesion reliability of the Geckus structure under real-world moist skin-attachment scenarios. The results show that the peak peeling force reaches approximately 0.53 N under ambient pressure and approximately 0.44 N under low pressure, indicating sufficient adhesion capability for skin-attachable electronic devices under diverse environmental conditions.


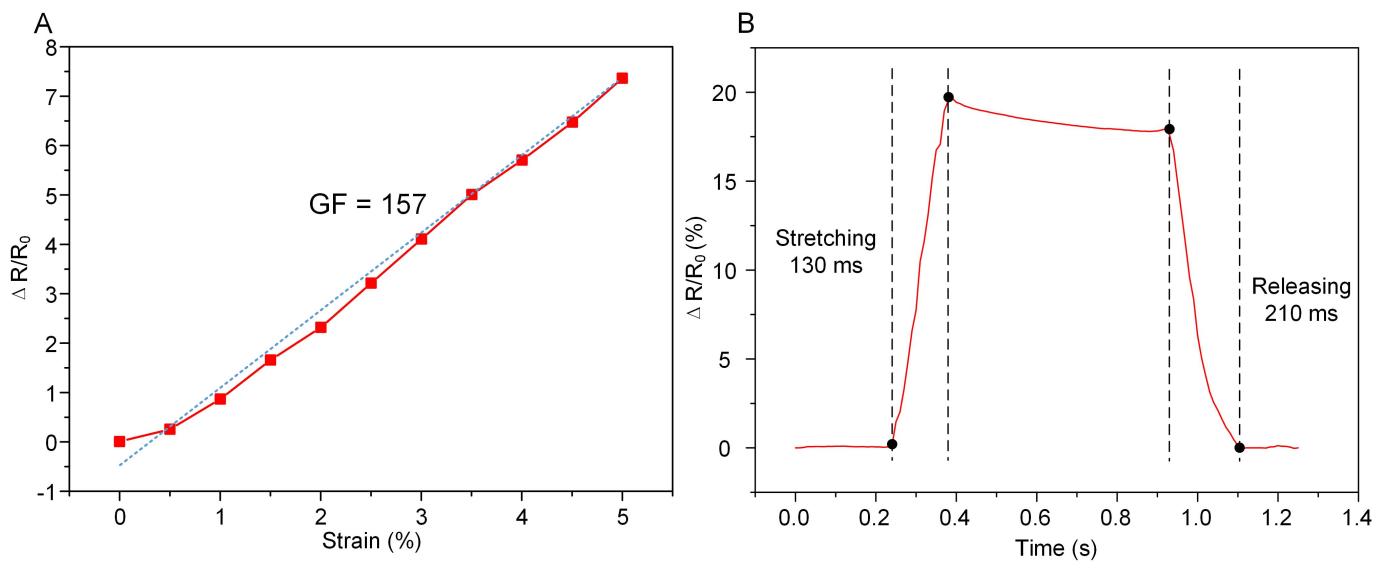


**Fig. S26.** Characterizations of the quantitative sensing metrics of the wearable device. (a) Gauge factor of the device. (b) Response time of the device. The sensor exhibits a gauge factor (GF) of 157, with response and recovery times of 130 ms and 210 ms, respectively, indicating good dynamic performance.

**
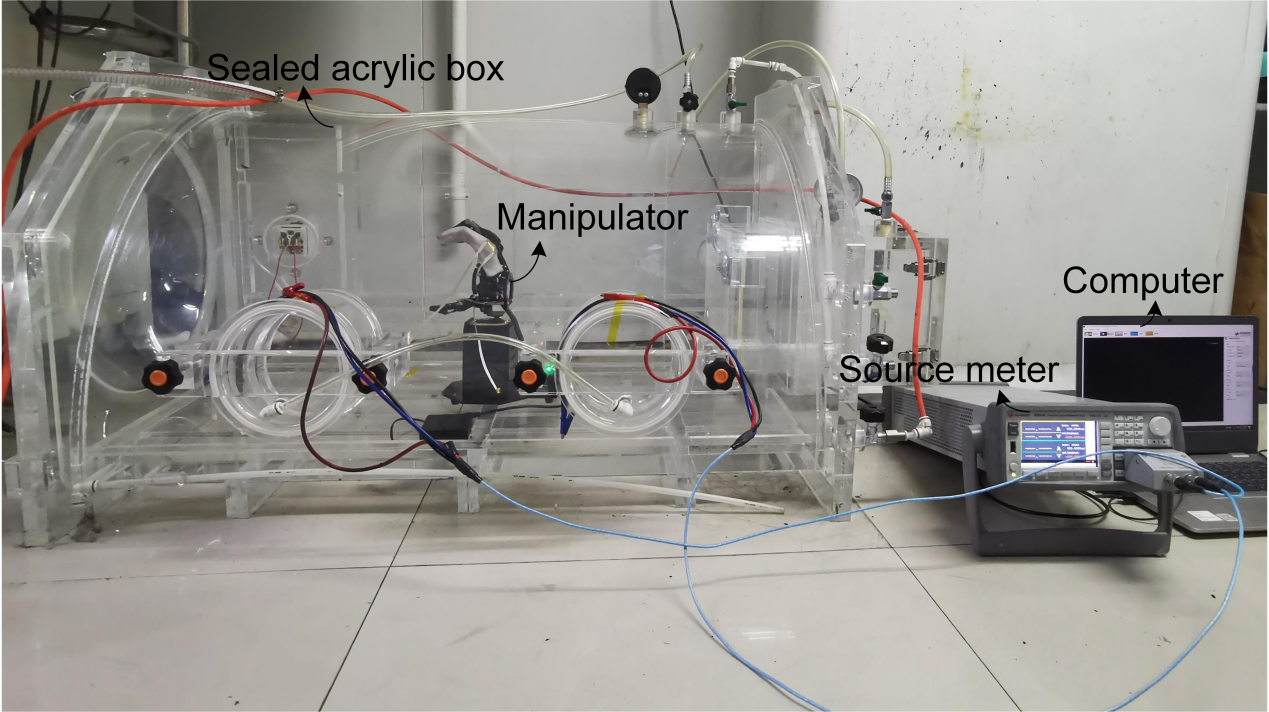
**

**Fig. S27.** Sensing method of the performances towards the skin-attachable device in vacuum (pressure of 30 kPa). The remote-controlled manipulator was placed in the sealed acrylic box to simulate the human hand in a low-pressure environment. The sensor signals are collected by the computer.

**
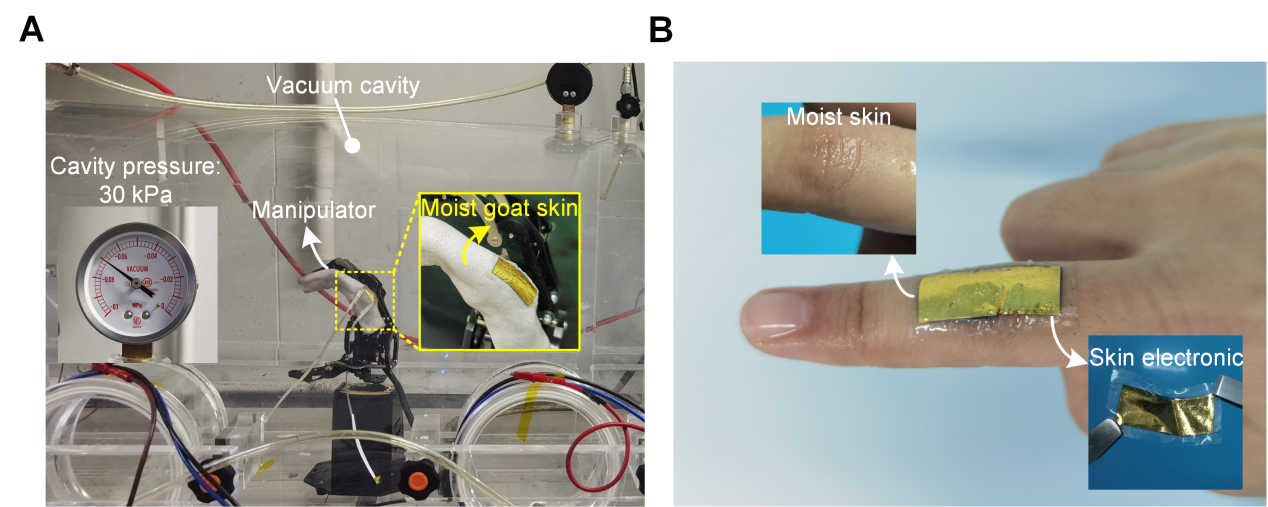
**

**Fig. S28.** Detailed information towards the applications of skin-attachable device. (A) In vacuum but moist goat skin surface. (B) In dry environment but moist human skin surface. The devices are tightly and conformally attached to the moist skin surfaces, whether in the vacuum or atmosphere conditions, which are both the typical application scenarios of multiple environments.


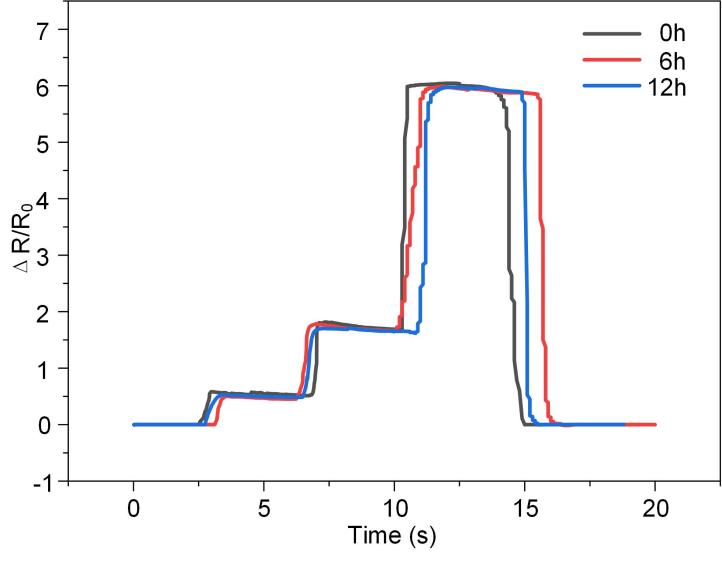


**Fig. S29.** Follow-up response of the device measured at 0 h, 6 h, and 12 h under different bending angles of the finger. The wearable electronics using the Geckus structure as the adhesive layer maintained a stable tracking response during 12 hours of continuous attachment, demonstrating that the Geckus adhesive possesses long-term attachment stability.


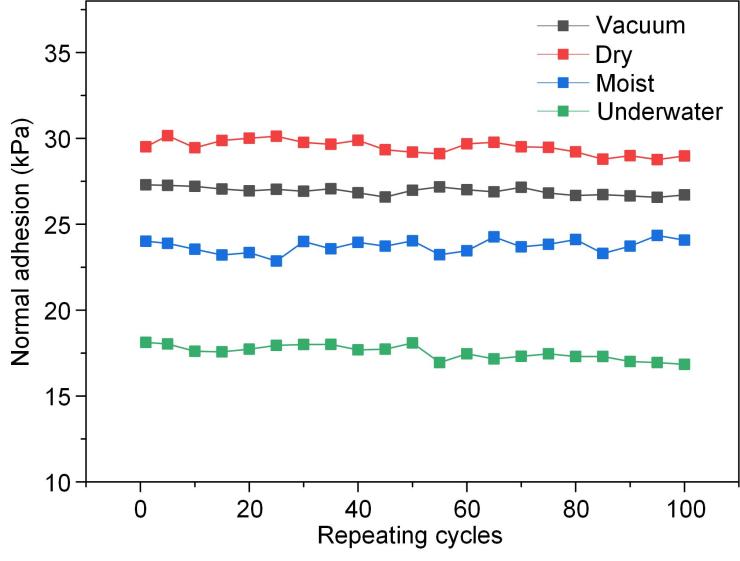


**Fig. S30.** Repeatability test of the skin-attachable device on goat skin under vacuum, dry, moist, and underwater conditions. The device exhibits considerable and reversible normal adhesion on the rough goat skin in vacuum, dry, moist, and underwater environments even after 100 cycles of attachment and detachment.


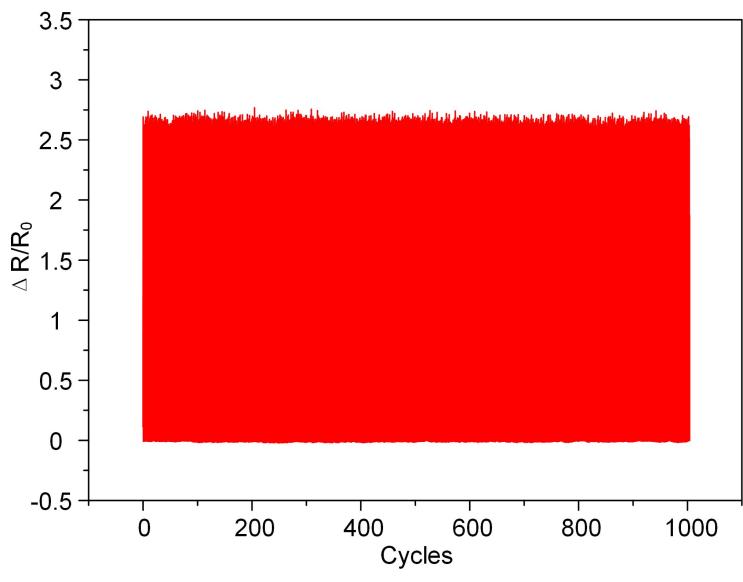


**Fig. S31.** Characterization of the bending repeatability of the skin-attachable device. After 1000 bending cycles, the resistance change rate of the device remained approximately 2.7, demonstrating the reliability of the device.

**Supplementary tables**

**Table S1.** Parameters used in numerical analysis of liquid filling

| Simulation parameters | Fluids | Value |
| --- | --- | --- |
| Mass density | Air | 1.29 kg/m^3^ |
| Mass density | Liquid | 1000 kg/m^3^ |
| Viscosity | Air | 1.79E-5 Pa·s |
| Viscosity | Liquid | 0.1 Pa·s |
| Surface tension coefficient | Air–liquid interface | 0.03 N/m |
| Contact Angle | Liquid to the solid | 65/180*pi |
| Contact Angle | Liquid to the solid | 70/180*pi |
| Contact Angle | Liquid to the solid | 80/180*pi |

**Table S2.** Pressure state of individual surfaces at different stages

| Stage | Press-Inner Surface Pressure (Pa) | Press-Outer Surface Pressure(Pa) | Press-Bot Surface Pressure(Pa) |
| --- | --- | --- | --- |
| Initial | 1.013×105 | 1.013×105 | 1.013×105 |
| Preload | $V_{Inner}$ | 1.013×105 | 0 |
| Pull-1 | $V_{Inner}$ | 1.013×105 | 0 |
| Pull-2 | $V_{Inner}$ | 1.013×105 | $V_{Inner}$ |
| End | 1.013×105 | 1.013×105 | 1.013×105 |

**Table S3.** Structural optimization parameters

| **Structural optimization parameters** | **Sizes** | | | | | |
| --- | --- | --- | --- | --- | --- | --- |
| *t* | 6 | 7 | 8 | 9 | 10 | 11 |
| *L_t_* | 13 | 15 | 20 | 25 | 30 | 35 |

**Table S4.** Comparison of normal adhesion performance of Geckus and other typical structures

| Structure Type | Environmental Condition | Normal  Adhesion (kPa) | Substrate  Surface | Material |
| --- | --- | --- | --- | --- |
| Geckus | Vacuum | 120 | Silicon wafer | PDMS |
|  | Dry | 201 |  |  |
|  | Moist | 165 |  |  |
|  | Underwater | 173 |  |  |
| OIAs | Dry | 25 | Silicon wafer | s-PUA |
|  | Moist | 37 |  |  |
|  | Underwater | 41 |  |  |
| AOC | Dry | 66 | Silicon wafer | PDMS |
|  | Moist | 53 |  |  |
|  | Underwater | 45 |  |  |
| μ-SC | Dry | 30 | Silicon wafer | s-PUA |
|  | Underwater | 110 |  |  |
| EOMS | Dry | 86 | Silicon wafer | PDMS |
|  | Underwater | 61 |  |  |
| EDA | Vacuum | 80 | Glass | Carbon black-doped PDMS |
|  | Dry | 100 |  |  |
| DIA | Dry | 43 | Silicon wafer | PDMS |
|  | Underwater | 35 |  |  |

**Supplementary movies**

**Movie S1.** Transporting 1 kg weight in vacuum, dry, moist and underwater environments.

**Movie S2.** The contact-splitting behavior of EOMS in dry conditions.

**Movie S3.** The contact-splitting behavior of MSAM in dry conditions.

**Movie S4.** The contact-splitting behavior of Geckus in dry conditions.

**Movie S5.** The contact-splitting behavior of MSAM in underwater conditions.

**Movie S6.** The contact-splitting behavior of EOMS in underwater conditions.

**Movie S7.** The contact-splitting behavior of Geckus in underwater conditions.
